# Can we diagnose mental disorders in children? A large‐scale assessment of machine learning on structural neuroimaging of 6916 children in the adolescent brain cognitive development study

**DOI:** 10.1002/jcv2.12184

**Published:** 2023-06-28

**Authors:** Richard Gaus, Sebastian Pölsterl, Ellen Greimel, Gerd Schulte‐Körne, Christian Wachinger

**Affiliations:** ^1^ The Lab for Artificial Intelligence in Medical Imaging (AI‐Med) Department of Child and Adolescent Psychiatry Ludwig‐Maximilians‐Universität Munich Germany; ^2^ Department of Child and Adolescent Psychiatry, Psychosomatics and Psychotherapy University Hospital Ludwig‐Maximilians‐Universität Munich Germany; ^3^ Department of Radiology Technical University of Munich School of Medicine Munich Germany

**Keywords:** ABCD study, confounding, machine learning, mental disorders, neuroimaging

## Abstract

**Background:**

Prediction of mental disorders based on neuroimaging is an emerging area of research with promising first results in adults. However, research on the unique demographic of children is underrepresented and it is doubtful whether findings obtained on adults can be transferred to children.

**Methods:**

Using data from 6916 children aged 9–10 in the multicenter Adolescent Brain Cognitive Development study, we extracted 136 regional volume and thickness measures from structural magnetic resonance images to rigorously evaluate the capabilities of machine learning to predict 10 different psychiatric disorders: major depressive disorder, bipolar disorder (BD), psychotic symptoms, attention deficit hyperactivity disorder (ADHD), oppositional defiant disorder, conduct disorder, post‐traumatic stress disorder, obsessive‐compulsive disorder, generalized anxiety disorder, and social anxiety disorder. For each disorder, we performed cross‐validation and assessed whether models discovered a true pattern in the data via permutation testing.

**Results:**

Two of 10 disorders can be detected with statistical significance when using advanced models that (i) allow for non‐linear relationships between neuroanatomy and disorder, (ii) model interdependencies between disorders, and (iii) avoid confounding due to sociodemographic factors: ADHD (AUROC = 0.567, *p* = 0.002) and BD (AUROC = 0.551, *p* = 0.002). In contrast, traditional models perform consistently worse and predict only ADHD with statistical significance (AUROC = 0.529, *p* = 0.002).

**Conclusion:**

While the modest absolute classification performance does not warrant application in the clinic, our results provide empirical evidence that embracing and explicitly accounting for the complexities of mental disorders via advanced machine learning models can discover patterns that would remain hidden with traditional models.

**Key points**

- Prediction of mental disorders based on neuroimaging showed promising results in adults.
- It is doubtful whether findings obtained on adults can be transferred to children.
- We carry out an explorative analysis to rigorously evaluate the potential of neuroimaging and machine learning to predict ten different psychiatric disorders in an ecologically valid sample of 6916 children from the Adolescent Brain Cognitive Development (ABCD) study.
- Our results indicate that attention deficit hyperactivity disorder (ADHD) and bipolar disorder (BD) can be predicted with statistical significance if one accounts for the complexities of mental disorders.
- While the overall low classification performance does not warrant application in the clinic, our results highlight that future studies should apply advanced machine learning models that are appropriate for the task at hand.

## INTRODUCTION

A growing body of research focuses on using machine learning methods to identify viable neuroimaging biomarkers for mental illnesses (Arbabshirani et al., 2017; Jollans & Whelan, 2018; Rashid & Calhoun, 2020; Sakai & Yamada, 2019; Sui et al., 2020; Wolfers et al., 2015; Woo et al., 2017). Such biomarkers could help to stratify patients according to different prognoses, thereby allowing psychiatric treatments to be tailored to individual patients and to improve in efficacy (Bzdok & Meyer‐Lindenberg, 2018; Kapur et al., 2012). The most successful studies employing machine learning are those focusing on Parkinson's disease, Alzheimer's disease, schizophrenia, major depressive disorder (MDD), autism spectrum disorder, and ADHD (Arbabshirani et al., 2017; Sakai & Yamada, 2019; Woo et al., 2017). While positive results can be expected for neurodegenerative disorders that involve neuroanatomical changes visible on a macroscopic level (Love et al., 2018), it is notable that similar results were shown for mental disorders for which distinct disease‐causing neuroanatomical changes have not been established (Borsboom et al., 2019). For instance, for MDD, median reported accuracies are as high as 79% (Sakai & Yamada, 2019), 82% (Arbabshirani et al., 2017), and 86.7% (Woo et al., 2017), while sensitivity and specificity are within the 70%–90% range (Kambeitz et al., 2017). Promising results are also reported by a few studies exclusively focusing on mental disorders in child and adolescent participants (First et al., 2018). Using structural magnetic resonance imaging (sMRI) data from the ENIGMA consortium, ADHD in children could be detected with a test set area under the curve of 0.64 (Zhang‐James et al., 2021). Pediatric depression could be successfully predicted with an accuracy of 78.4% (Wu et al., 2015). However, these previous findings are limited in their generalizability by the fact that small‐scale studies that investigate single disorders exclusively in adults still dominate. In particular, research on neuroimaging biomarkers in children is rare and it is doubtful whether biomarker findings obtained on adults can be readily transferred to children.

Building on the ABCD study (Karcher & Barch, 2021) and the ABCD Neurocognitive Prediction Challenge (Pohl et al., 2019), we seek to carry out an explorative analysis to rigorously evaluate the potential of using sMRI and machine learning techniques to predict 10 different psychiatric disorders in an ecologically valid sample of 6916 children. We study MDD, BD, psychotic symptoms, ADHD, ODD, conduct disorder (CD), post‐traumatic stress disorder, obsessive‐compulsive disorder (OCD), generalized anxiety disorder (GAD), and social anxiety disorder (SAD).

## METHODS

### Participants

**FIGURE 1:**
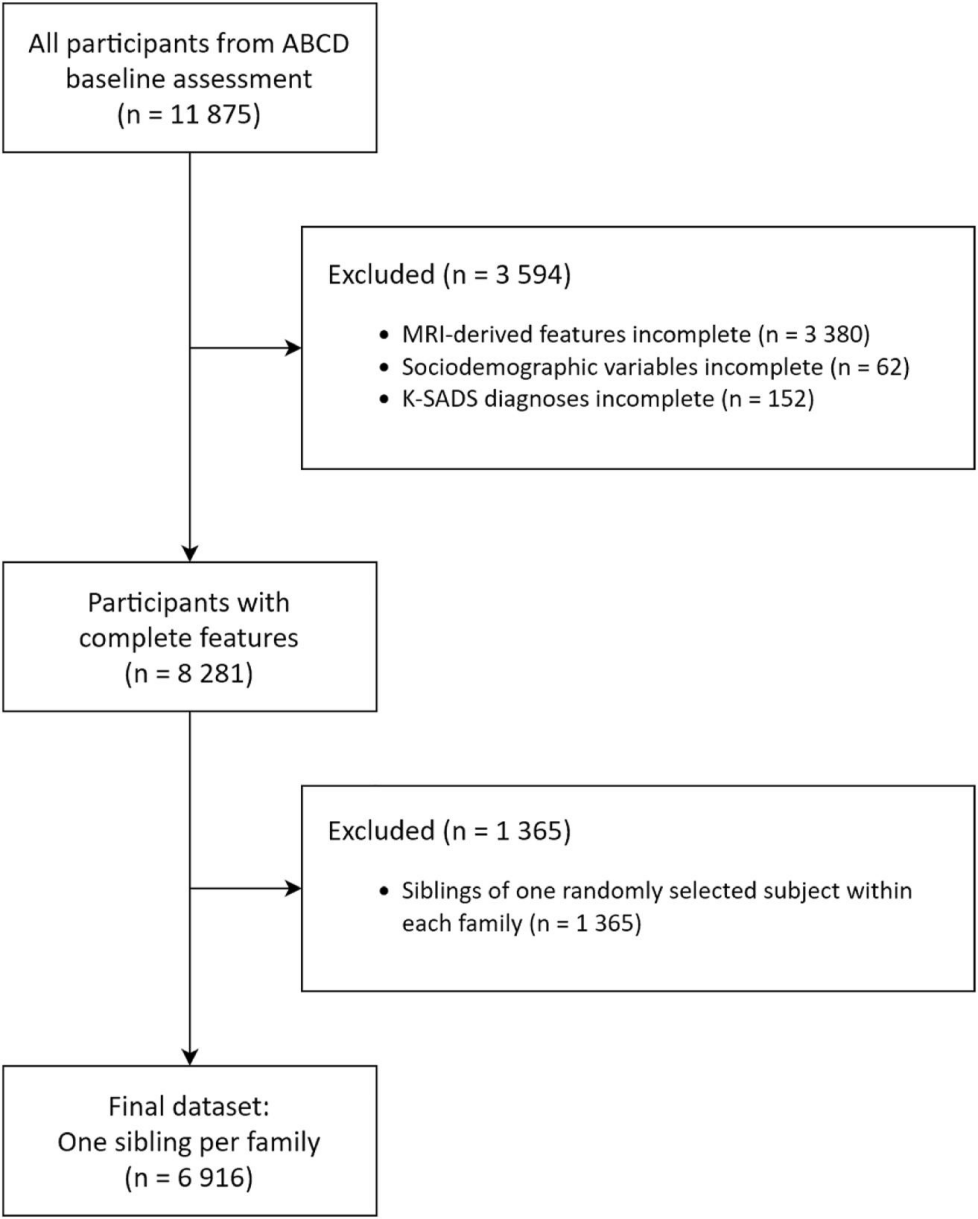
Flow diagram of participant selection.

All 11,875 participants from the baseline assessment of the ABCD study (Karcher & Barch, 2021) were considered for inclusion in the present study. The participants of the ABCD study were mainly recruited through the US school systems. Adolescent Brain Cognitive Development focused on ensuring that the sample reflects the diversity of the US population by employing probability sampling of US schools as the primary method for recruiting eligible children. To this end, school selection was informed by gender, race and ethnicity, socioeconomic status, and urbanicity. Despite the effort in ABCD to match the demographics of the US population, it may not be representative in all dimensions that influence a child's development. Participants provided informed consent (parents) and assent (child). First, all participants with missing structural brain measures (see section Imaging Data below) were excluded. Imputation is not an option for recovering missing image features as either all or none of the brain measures are available. Second, we also excluded all participants for which Kiddie Schedule for Affective Disorders and Schizophrenia (K‐SADS) diagnoses for the investigated clinical conditions were missing. To rule out any statistical dependencies due to sibling relationships from the data, we excluded all but one randomly selected child from each family. For a detailed flow diagram of the participant selection, see Figure 1.

### Psychiatric disorders

The psychiatric disorders in the ABCD study were diagnosed using a computerized version of the K‐SADS (Kaufman et al., 1997) for DSM‐5 (KSADS‐5; see (Barch et al., 2018) for details regarding the application of this instrument in the ABCD study). The paper‐and‐pencil KSADS is a well‐established diagnostic interview with good to excellent test‐retest reliability (Cohen's Kappa = 0.63–1.00) (Ambrosini, 2000) and high interrater agreement (93%–100%) (Kaufman et al., 1997). Preliminary validity data on the computerized KSADS‐5 demonstrated that it possesses good to excellent clinical validity (Townsend et al., 2020). Initially, we considered all DSM‐5 coded diagnoses (American Psychiatric Association, 2013) that were available in the baseline assessment of the ABCD study (see Table S2 from (Barch et al., 2021)) as prediction targets. From these, we excluded all “unspecified” and “other specified” diagnoses due to validity concerns and the diagnoses for persistent depressive disorder, panic disorder, agoraphobia, eating disorders, disruptive mood dysregulation disorder, and substance use disorder due to sparse case numbers (Table S1). Separation anxiety disorder was excluded, because two common anxiety disorders, generalized and SAD, were already included.

Most psychiatric diagnoses in the ABCD study were assessed by both parental and youth self‐report. A well‐documented phenomenon in such multi‐informant mental health assessments is high rates of disagreement between parent and self‐reports (De Los Reyes et al., 2015), with the ABCD study being no exception. While historically many techniques have been proposed to integrate these discrepant reports into a best estimate of the child's “true” diagnostic state, there exists no established best practice so far (Martel et al., 2017). A frequently applied—prominently in the DSM‐IV field trials (Lahey et al., 1994)—and robust (Bird et al., 1992; Piacentini et al., 1992) strategy is the OR rule where a child's symptom is considered present when it is reported by at least one informant. We used the OR rule to aggregate parental and self‐report DSM‐5 diagnoses into a single binary disorder for each child. Moreover, we assumed the presence of a disorder when the diagnosis was given at any time state (current, past, and in remission) to capture not only the current state but the lifetime history of the disorder.

To increase case numbers, we aggregated bipolar I and II disorder into a composite category “bipolar disorder”. Lastly, while there is robust evidence for brain structural abnormalities in schizophrenia (van Erp et al., 2018), the baseline assessment of the ABCD study did not include any diagnoses from the Schizophrenia Spectrum and Other Psychotic Disorders DSM‐5 category. To still include a surrogate of psychotic symptomatology in our study, we defined “psychotic symptoms” as the presence of at least one of the symptoms of hallucinations, delusions, or associated psychotic symptoms or a diagnosis of unspecified schizophrenia spectrum/other psychotic disorder in the KSADS‐5 parent interview.

### Imaging data

To be able to capture a diverse range of anatomical changes, we extracted 136 volumetric and thickness measures based on FreeSurfer (Fischl, 2012) (see Table S2) from participants' T1‐weighted sMRI. See Appendix S1 for a detailed description of the feature extraction pipeline. We do not include functional MRI data in the analysis due to the very poor reliability of task‐related brain activations in the ABCD sample (Kennedy et al., 2022), largely preventing its use for studying individual differences.

### Known confounders

To account for known confounding effects on the relationship between neuroanatomy and disorder, we residualized (see Appendix S1) all 136 neuroanatomical measures to exclude effects due to the sociodemographic variables age, sex, marriage status of parents, study site, highest parental education, ethnicity, and supratentorial brain volume, which were used as known confounders on fluid intelligence in the ABCD Neurocognitive Prediction Challenge (Pohl et al., 2021).

### Machine learning models

We trained ensembles of gradient boosted trees (GBM; (Friedman, 2001);) to predict the binary psychiatric disorder based on brain structural measures. GBMs currently represent the state‐of‐the‐art in classification algorithms (Zhang et al., 2017). Bayesian hyperparameter optimization (J. Snoek et al., 2012) was used to tune the hyperparameters of the model. To account for interdependencies between disorders, we constructed classifier chains (Read et al., 2011) of 10 GBMs. For each classifier chain, disorders are randomly ordered in a list and 10 GBMs are trained, where the *i*th GBM is trained to predict the *i*th disorder in the list, using image‐derived measurements and the presence/absence of all disorders preceding *i* in this list. We considered multiple such random orderings by creating a final ensemble of 10 such chains, which we refer to as GBM‐CCE. In addition, we employed a simple logistic regression classifier (LRC) as a linear benchmark model to compare to. See Appendix S1 for a detailed description of the machine learning models.

### Model evaluation

**FIGURE 2:**
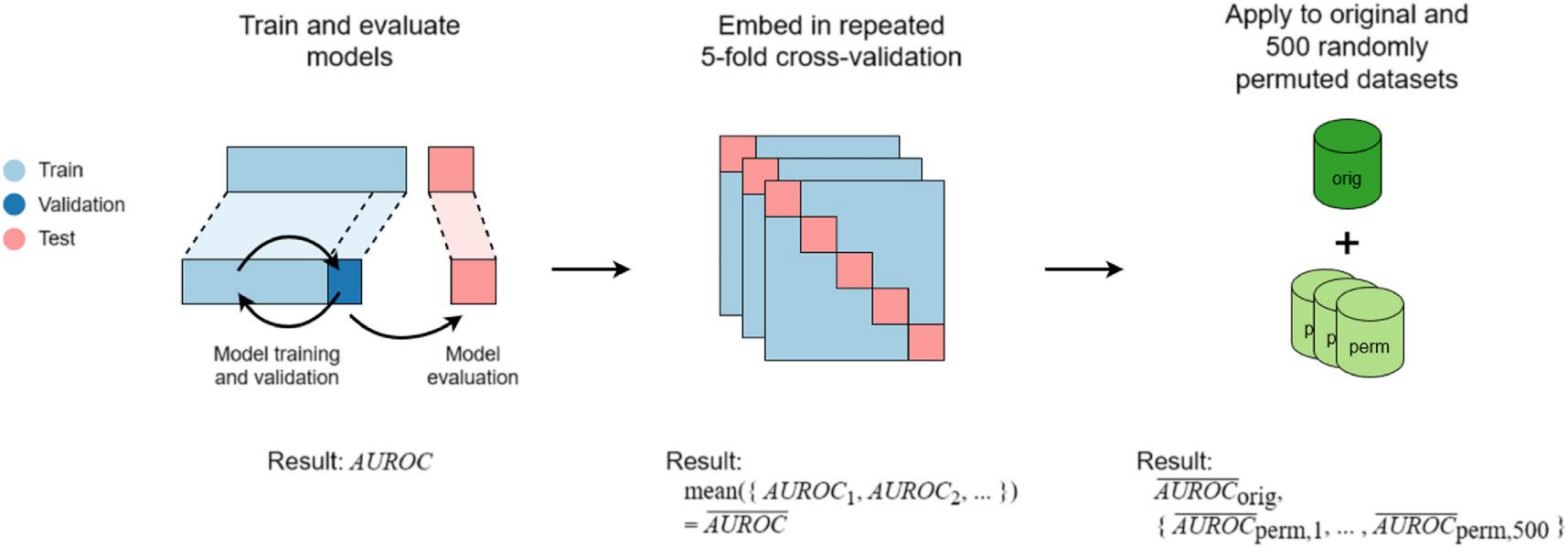
Model evaluation pipeline. Area under the receiver operating characteristic curve (AUROC) values are obtained by evaluating trained models on the test set, then averaged over all cross‐validation folds. This results in one average AUROC for the original dataset and a distribution of 500 average AUROC representing the distribution under the null hypothesis of “no real pattern has been discovered”.

The performance of the GBM‐CCE and LRC in predicting each disorder in the test set was measured in terms of the area under the receiver operating characteristic curve ( AUROC). To assess statistical robustness and reproducibility, we employed a 30‐times repeated 5‐fold cross‐validation scheme (see Figure 2). At every one of the 30 repeats of the outer loop, the dataset was randomly divided into five parts of equal size (folds). For each of the five repeats of the inner loop, model training and validation were performed on four of the folds (80% of the data), and the model was tested on the remaining fold (20%) until each fold has been used as the test set exactly once. Each of these 150 individual cross‐validation splits represented an independent experiment with a newly initialized model, so that there was no data leakage. All data splits were stratified with respect to all eight disorders to ensure homogeneous label distributions between splits. Finally, the resulting 150 unique test set AUROC values were averaged. In addition, we calculated mean balanced accuracy (Brodersen et al., 2010), mean sensitivity, and mean specificity based on the binary classification thresholds corresponding to the highest Youden's J statistic (Youden, 1950) in each individual test set.

To statistically test whether the models had found real patterns in the features that are predictive of a particular disorder, we employed the permutation test described in (Ojala & Garriga, 2010), which yielded a *p*‐value for each model with the null hypothesis that the model did not discover a meaningful relationship between features and labels (see Appendix S1 for details). As we performed 20 independent permutation tests (two models times 10 disorders), we applied Bonferroni correction to obtain the adjusted significance level $\alpha_{adj}=\frac{0.05}{20}=0.0025$.

## RESULTS

### Sample characteristics

**FIGURE 3:**
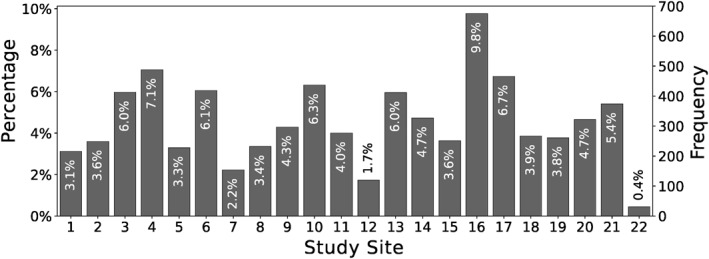
Participants per geographical study site.

**FIGURE 4:**
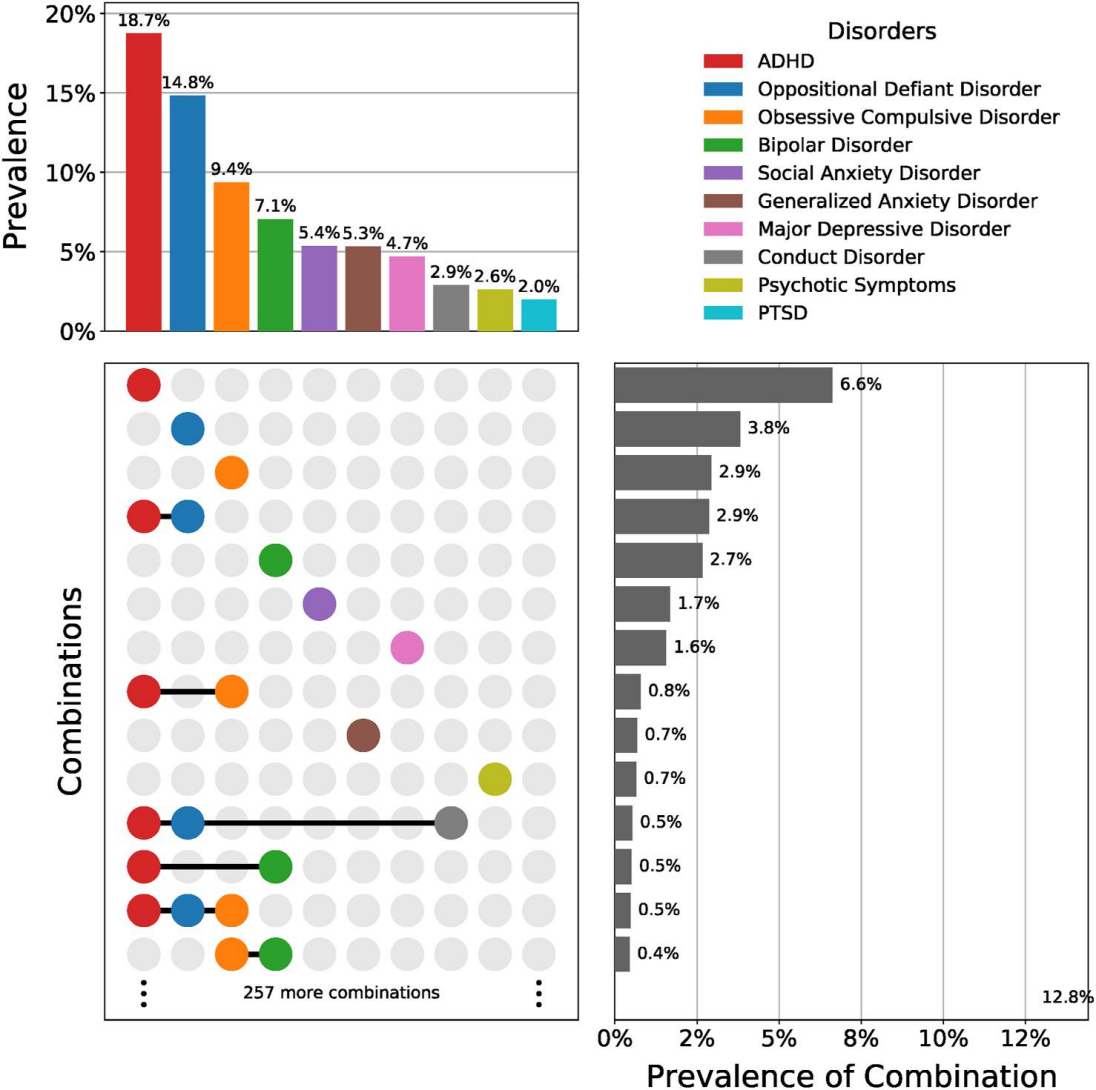
Comorbidities of studied mental disorders. (top) Studied mental health conditions with overall prevalence; (bottom) 14 most common patterns of disorders and their prevalence. Having no disorder at all was most common (63.7% of participants).

We used a sample of 6916 children from the ABCD study (Karcher & Barch, 2021) (see Figure 1). Table 1 summarizes the characteristics of the sample used in our study. Data was pooled from 22 study sites with a median of 4.0% (IQR: 3.4%–6.0%) of participants coming from a single site (see Figure 3). The most common disorder was ADHD with a prevalence of 18.7% (see Figure 4, top), followed by ODD (14.8%), and OCD (9.4%). The most common co–occurrence of disorders was that of ADHD and ODD with a prevalence of 2.9%, which was more common than any isolated disorder, except for isolated ADHD or ODD (see Figure 4, bottom).

**TABLE 1:** Demographics of selected participants.

Measure	Statistic
Age	9.9 (0.6) years
Gender	
Female	3617 (47.7%)
Male	3299 (52.3%)
Race/Ethnicity	
Asian	162 (2.3%)
Black	761 (11.0%)
Hispanic	1464 (21.2%)
White	3834 (55.4%)
Other	695 (10.0%)
Marriage status of parents	
Married	4820 (69.7%)
Unmarried	2096 (30.3%)
Highest parental education	
Less than high‐school diploma	272 (3.9%)
High‐school diploma or GED	564 (8.2%)
Some college	1782 (25.8%)
Bachelor's degree	1821 (26.3%)
Post‐graduate degree	2477 (35.8%)

*Note*: Data are counts and percentage in parenthesis, except for Age, where mean and standard deviation are provided.

### Predictive performance

**FIGURE 5:**
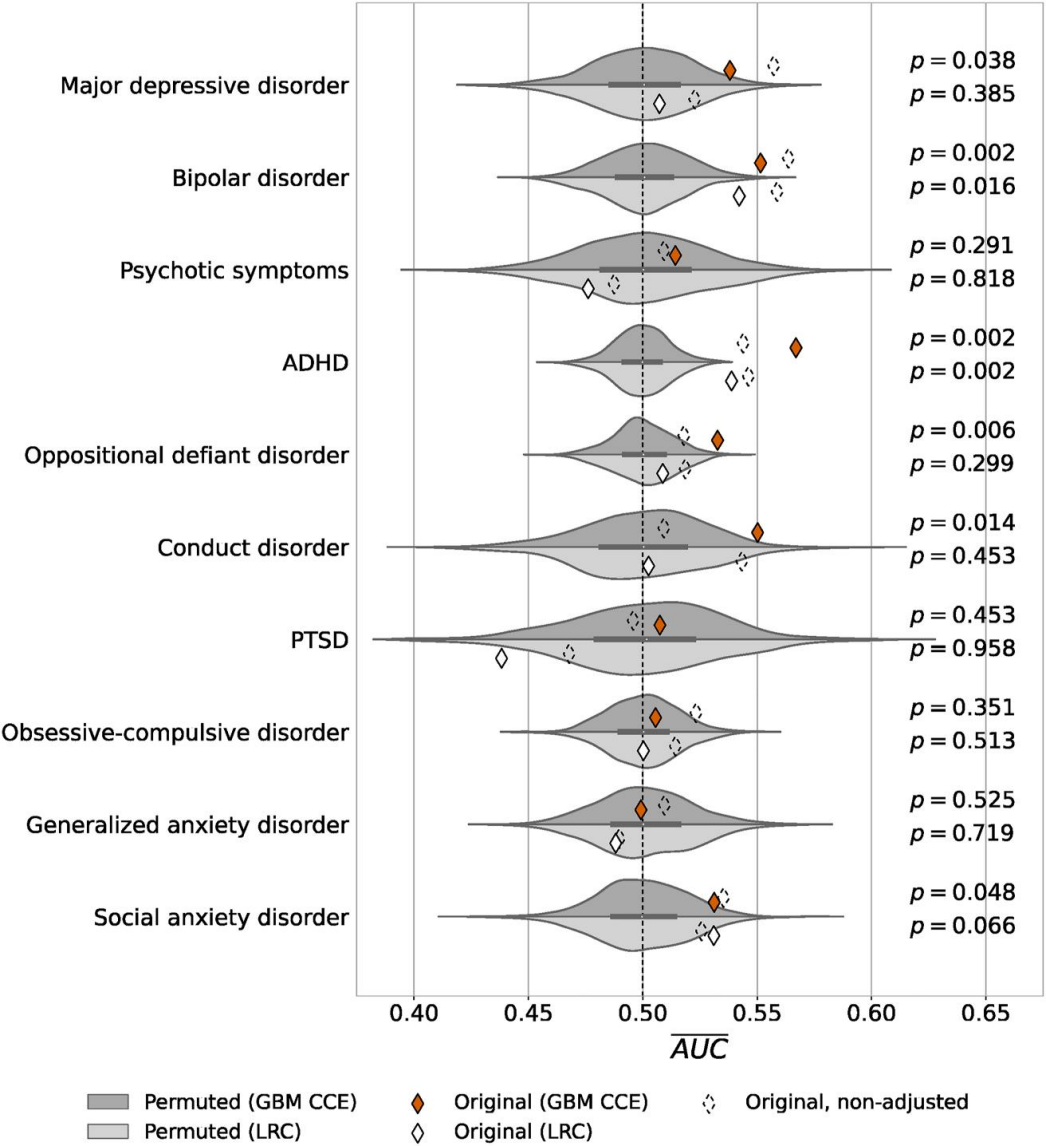
Violin plots of cross‐validation results. For each disorder and both classifiers, the distribution of area under the receiver operating characteristic curve (AUROC) under the nullhypothesis of “no real pattern has been discovered” (in gray) is contrasted with the AUROC value (diamond) on the original dataset. Dashed diamonds show AUROC values on unpermuted data with no adjustment by sociodemographic confounders (see Table S5 for a statistical comparison with original AUROC values). Dashed line at $\bar{AUROC}=0.5$ corresponds to a classifier with no discriminative ability. CCE, Gradient boosting model classifier chain ensemble; GBM‐LRC, Logistic regression classifier.

We studied the predictive performance of detecting each of the binary psychiatric disorders using 136 regional subcortical volumes and cortical volume and thickness measurements derived from sMRI. Using an ensemble of classifier chains of gradient boosted trees (GBM‐CCE) has two major advantages over a traditional LRC. First, by relying on gradient boosted trees (Friedman, 2001), we do not have to specify non‐linear feature transformations nor interaction terms explicitly, but can learn those implicitly from the data. Second, by using classifier chains (Read et al., 2011), our model considers all 10 disorders concurrently. Specifically, we use classifier chains to leverage interdependencies among disorders, which is particularly relevant due to the high prevalence of comorbidities among mental illnesses in youth (Newman et al., 1998). Moreover, we accounted for confounding due to acquisition site and sociodemographic factors by residualizing MRI‐derived measurements. Figure 5 summarizes the predictive performance in terms of the AUROC for all 10 disorders, and whether the model has found a real pattern in the data based on permutation testing. Finally, we repeated our experiments using volumetric measures based on the SRI24 Atlas (Rohlfing et al., 2010). Results for these experiments are summarized in Appendix S2.

The GBM‐CCE achieved a statistically significant prediction performance for two disorders: ADHD (mean AUROC = 0.567, *p* = 0.002) and BD (mean AUROC = 0.551, *p* = 0.002). For both disorders, the mean AUROC was higher than all 500 AUROC values on the permuted data sets, resulting in the lowest possible *p*‐value of $p=\frac{1}{500}=0.002$. Table 2 provides the mean balanced accuracy, sensitivity and specificity for these disorders. Mean balanced accuracy was 56.1% and 56.2%, mean sensitivity 57.7% and 52.6%, and mean specificity 54.6% and 59.9% for ADHD and BD, respectively. The two disorders, which were predicted significantly, were also among the four most prevalent.

**TABLE 2:** Disorders for which significant predictions were achieved.

		Disorders	
		ADHD	Bipolar disorder
Best model		GBM‐CCE	GBM‐CCE
AUROC	Mean	0.567	0.551
	Range	0.523–0.608	0.487–0.620
Balanced accuracy	Mean	56.1%	56.2%
	Range	53.3%–59.7%	51.5%–61.0%
Sensitivity	Mean	57.7%	52.6%
	Range	30.7%–89.3%	13.8%–93.1%
Specificity	Mean	54.6%	59.9%
	Range	20.0%–80.3%	17.8%–93.6%

*Note*: Shown are mean and range of 150 different test set AUROC values obtained by 30‐times repeated 5‐fold cross‐validation on the original dataset. Mean of balanced accuracy, sensitivity, and specificity correspond to the maximum Youden's J statistic.

To investigate the performance improvement due to modeling interdependencies among disorders, and non‐linear relationships, we evaluated a simple LRC that does not have these desirable properties. As seen in Figure 5, such a model achieved a statistically significant prediction only for ADHD, and with a lower performance (AUROC = 0.539, *p* = 0.002).

### Impact of confounding

Finally, we evaluated the impact of confounding factors by fitting LRC and GBM‐CCE models on the original volume and thickness measurements without adjusting them for confounding factors via residualization (see dashed diamonds in Figure 5). The results demonstrate that ignoring confounding resulted in an inflated prediction performance in five out of 10 disorders for the GBM‐CCE: MDD, BD, OCD, GAD, and SAD. Whereas for LRC, ignoring confounding inflated the predictive performance for all disorders except SAD. The difference in performance with and without adjustment for confounding was highly significant in seven out of 10 disorders for both the GBM‐CCE and the LRC (Table S5).

## DISCUSSION

In this study, we evaluated the potential of structural neuroimaging to detect 10 psychiatric disorders in children, based on T1‐weighted MRI from 6916 children from the ABCD study (Karcher & Barch, 2021). Compared to many earlier studies, the ABCD study offers the advantage of an exceptionally high degree of heterogeneity and ecological validity due to pooling data from 22 sites and featuring mental disorder prevalence and comorbidity rates that closely approximate that of the general population. Moreover, the ABCD study includes assessments of many sociodemographic measures, which enables a rigorous analysis of confounding effects.

Our evaluation showed that the GBM‐CCE was able to learn statistically significant patterns to detect ADHD and BD. These findings are suggestive of the existence of brain structural patterns that are associated with certain mental disorders and corroborate previous findings (Arbabshirani et al., 2017; Rashid & Calhoun, 2020; Sakai & Yamada, 2019; Wolfers et al., 2015; Woo et al., 2017). Importantly, we only obtained a statistically significant classification performance for BD when accounting for interdependencies and co‐occurrence of disorders, and non‐linear relationships between neuroimaging biomarkers and disorder. A traditional logistic regression model that does not account for interdependencies nor non‐linear relationships failed to achieve a statistically significant prediction performance for BD and showed lower classification performance in terms of AUROC for all disorders compared with the GBM‐CCE. First, this confirms that the relationship between brain structure and disorders is highly non‐linear (Arbabshirani et al., 2017). Second, it suggests that there is no one‐to‐one mapping between neuroanatomy and disorder, and that patterns of comorbidity can only be leveraged if appropriate machine learning models are employed. The latter relates to the fact the etiopathophysiology of psychiatric disease is highly complex due to "functional dependencies among neural systems that yield complex patterns of comorbidity” (Beauchaine & Hinshaw, 2020) (p. 329). Moreover, there is a growing concern that classifying subjects into discrete and seemingly independent categories does not align with underlying neurobiological mechanisms (Astle et al., 2022; Beauchaine & Hinshaw, 2020). Here, we acknowledged that symptoms are possibly an imperfect basis for psychiatric nosology and interdependencies exist, and employed machine learning models, namely classifier chain ensembles, that leverage interdependencies among symptoms. Our additional experiments based on neuroanatomical features from the SRI24 Atlas (see Appendix S2) confirmed that GBM‐CCE is often preferred over LRC (5 of 10 disorders)—although none of the results achieved statistical significance. This suggests that the choice of neuroanatomical segmentation algorithm may impact the maximum achievable performance, but that modeling interdependencies between diagnoses is often preferred, independent of the segmentation algorithm.

When repeating those analyses without confounding correction (see dashed diamonds in Figure 5), 14 out of 20 experiments resulted in inflated significance. These results indicate that the relationship between neuroanatomy and mental disorder is often confounded. This is confirmed by our supplementary analyses using the SRI24 Atlas, where 17 of 20 experiments were inflated. Hence, it is paramount to account for common confounders to minimize the risk that machine learning models leverage spurious correlations rather than biologically meaningful concepts in their predictions. Alternatively to residualization, one could control for confounding by including sociodemographic variables as additional predictors. While this will likely increase the overall prediction performance, it would not allow us to accurately evaluate the predictive performance of neuroimaging alone, because neuroimaging and sociodemographic factors will contribute to the prediction. Common sources of confounding in neuroimaging studies include imaging site, age, gender, and head size (Kirkpatrick et al., 2020; Nielsen et al., 2020; Scheinost et al., 2019; Wachinger et al., 2020). In addition, studies in children are often subject to bias due to sociodemographic factors. For instance, children in households with low parental education or those part of racial/ethnic minorities are less likely to participate in clinical research (Kirkpatrick et al., 2020; Reiss, 2013). In this study, we accounted for all of these factors to ensure reported prediction performances are indeed based on biologically meaningful signals.

The fact that two of the four most prevalent diagnoses (see Figure 4, top) could be predicted with statistical significance can in part be attributed to the variance of the AUROC under the nullhypothesis of “no real pattern has been discovered”. For a fixed sample size *n*, the variance of the AUROC will be larger for rare disorders compared to common disorders, because the variance of the AUROC scales inversely with *n*_1_⋅*n*_2_, where *n*_1_, *n*_2_ (*n* = *n*_1_ + *n*_2_) are the size of the number of cases and controls respectively (Hanley & McNeil, 1982). In turn, an increase in variance increases the threshold of statistical significance, which is evident from Figure 5 too. Hence, our results should be interpreted as conservative estimates with a focus on reducing the type I error (false positives).

While our findings are statistically significant, the low classification performance in absolute terms seems to contradict results from previous studies (Arbabshirani et al., 2017; Sakai & Yamada, 2019; Woo et al., 2017). Our highest achieved test set performance was a mean AUROC of 0.567 for ADHD (mean balanced accuracy of 56.1%). In contrast, the largest previous effort to predict ADHD in children (*N* = 2454) using sMRI features yielded an AUROC of 0.64 (Zhang‐James et al., 2021). Current reviews with a main focus on adults even report a mean classification accuracy of over 77% for ADHD across individual studies (Arbabshirani et al., 2017; Woo et al., 2017). For MDD, reviews report an accuracy of over 80% (Arbabshirani et al., 2017; Sakai & Yamada, 2019; Woo et al., 2017), which could not be predicted with statistical significance in our study. In the following, we highlight three key characteristics of the ABCD study that distinguish it from previous studies and that likely contribute to this marked discrepancy.

First, the recruitment process of the ABCD study ensures a near‐representative selection of participants to reduce systemic bias. It included probabilistic sampling of schools within the catchment of the 22 research sites (Garavan et al., 2018) and only minimal exclusion criteria (Thompson et al., 2019). Although the resulting sample should not be considered a fully representative sample (Compton et al., 2019), it closely matches the distribution of the U.S. population as a whole (Garavan et al., 2018), thus reaching an exceptionally high degree of ecological validity. In contrast, many of the previous studies assembled equally sized groups of affected and healthy participants (Arbabshirani et al., 2017; Zhang‐James et al., 2021).

The second factor that contributes to the observed discrepancy stems from the high heterogeneity that the ABCD studies captures and that is largely absent in previous studies. We distinguish between three sources of heterogeneity: (a) sociodemographic diversity, (b) comorbidities, and (c) scanning devices. (a) The ABCD study comprises a highly heterogeneous sample in terms of sociodemographic diversity, because children with a wide range of ethnical, cultural, and economic backgrounds were included. We accounted for these effects by residualizing the image‐derived measures. Our results indicate that confounding effects due to sociodemographic status can be substantial. For the two disorders that could be predicted with statistically significant performance, accounting for confounding effects increased the mean AUROC by 0.023 for ADHD and reduced it by 0.012 for BD (see dashed diamonds in Figure 5). (b) The second source of heterogeneity is due to the high rate of comorbidities (see Figure 4). Since different mental illnesses likely overlap in their neuroanatomical associations (Goodkind et al., 2015) and disorder subtypes can arise (Schnack & Kahn, 2016), accurate classification of subjects with comorbid clinical conditions is inherently more difficult. Nevertheless, the fact that our GBM‐CCE outperformed the LRC, shows that we can also leverage this overlap to improve accuracy. (c) Finally, the ABCD study is subject to heterogeneity due to scanning devices: it employed 29 different scanning devices by three different vendors, across 22 sites (Casey et al., 2018; Owens et al., 2021). Recent studies revealed that large multi‐center studies can be sensitive to confounding effects due to differences in acquisition, thus increasing heterogeneity in acquired scans and the measures extracted from them (Alfaro‐Almagro et al., 2021; Wachinger et al., 2020). How to best overcome heterogeneity due to differences in scanner is subject to ongoing research, and no consensus has been reached yet.

Precisely these three sources of heterogeneity are thought to be the reason why recent studies on neuroimaging for mental disorders found the striking result that classification accuracy consistently declines as the sample size of studies increases (Arbabshirani et al., 2017; Rashid & Calhoun, 2020; Sakai & Yamada, 2019; Wolfers et al., 2015; Woo et al., 2017). Smaller studies typically comprise a more homogeneous sample with fewer comorbidities, which in turn does away with many of the aforementioned challenges to discovering patterns for classifying the majority of participants accurately (Schnack, 2019). In this regard, our results are in line with our experience in the ABCD Neurocognitive Prediction Challenge (Pohl et al., 2019) where our team ranked third, but none of the predictions of fluid intelligence—of any team—was accurate enough to be meaningful (Pohl et al., 2019)—despite access to data from thousands of children.

The third factor that contributes to the observed discrepancy stems from the fact that our work was conducted exclusively on children. Firstly, diagnoses are more difficult to establish in children, because reports from multiple informants must be considered, and there is no single established best practice for aggregating them (Martel et al., 2017). In clinical practice, diagnoses are often given by mental health professionals. However, when only informant survey reports are available, as in the ABCD study, we must resort to simpler rule‐based aggregation—like the OR rule—which may not replicate the clinician gold standard in all cases. Secondly, the neuroanatomy of children's brains changes continuously and non‐linearly during maturation, and children of the same ages can be at different points in this process (Tamnes et al., 2017). This change may obscure structural neuroimaging patterns associated with psychiatric disorders, complicating accurate prediction.

In light of the modest classification performance, we currently do not see a clinical utility of biomarkers derived from structural MRI for the diagnosis of mental disorders in children. This is in line with the conclusion drawn by (First et al., 2018), who stated that neuroimaging has yet to make an impact on the diagnosis of psychiatric disorders in a clinical setting. Notably, this statement includes the diagnosis of adults, where prior research studies have reported high classification accuracy. The reason for the lacking clinical utility in classifying individuals is mainly due to the small effect sizes of neuroimaging markers with respect to psychiatric illnesses, yielding insufficient sensitivity and specificity. As a future direction, the combination of neuroimaging markers with non‐imaging data may be promising to improve the performance by establishing a more comprehensive picture of a patient. In this regard, exploring how neuroimaging can enhance already established diagnostic tools such as clinical and neurocognitive tests will be of particular clinical relevance.

There are several limitations to this study. First, because the ABCD study was conducted in the U.S., the data are limited to this geographic region. Factors such as symptom reports and ethnographic backgrounds may not be representative of other geographic and cultural contexts. Second, socioeconomic variables in the ABCD study are not exactly representative of the comparable‐age U.S. population. Both family income and the proportion of participants with married parents, who are both employed, are higher in the ABCD cohort (Heeringa & Berglund, 2020). Although we adjusted our analysis for parental marriage status, residual confounding may have persisted and may limit the generalizability of our results to the general population. Although we followed the common approach of assuming a linear relationship between confounding factors and neuroanatomical measures (L. Snoek et al., 2019), we cannot prove based on data alone that this is indeed the true (unknown) causal relationship. Moreover, the classifier is dependent on hyperparameters and while we have performed Bayesian hyperparameter optimization, further tuning may improve results. While model explainability was not an explicit focus of our study, future research on predicting mental disorders using neuroimaging could benefit from incorporating feature importance analyses, as this would add knowledge of the associations between features (such as regional brain volumes or comorbid disorders) and the target disorder. Finally, we captured neuroanatomical changes by relying on regional volume and thickness measures. While these measures are relatively robust to imaging noise, they cannot capture the full range of neuroanatomical changes, because multiple geometric structures can have the same volume/thickness. Hence, our models may fail to capture subtle changes in neuroanatomy.

## CONCLUSION

Our findings illustrate that detecting psychiatric disorders in children based on structural neuroimaging remains a significant challenge when generalization to large, ecologically valid, and heterogeneous samples is desired. At the same time, we showed that we can leverage comorbidities and interdependencies among symptoms to significantly improve prediction accuracy, although the absolute performance remains modest. To overcome common pitfalls, we argue that researchers should (i) study a heterogeneous sample, (ii) employ advanced machine learning techniques appropriate for the task at hand, and (iii) account for confounding effects due to sociodemographic factors. We analyzed a sample of 6916 children from the ABCD study (Karcher & Barch, 2021), which is the largest and most comprehensive study on psychiatric disorders in children to date. Next, we embraced the complexity of detecting psychiatric disorders by leveraging the capabilities of advanced machine learning models that are better suited for this task than traditional linear models. Finally, we ensured that our models were not significantly impacted by spurious correlations due to common sociodemographic factors by residualizing neuroanatomical measurements. We hope these strategies can form the basis for a push to advance our understanding of the etiopathophysiology of psychiatric disorders.

## AUTHOR CONTRIBUTION

**Richard Gaus**: Data curation; Methodology; Software; Validation; Visualization; Writing – original draft. **Sebastian Pölsterl**: Data curation; Methodology; Software; Writing – original draft; Writing – review & editing. **Ellen Greimel**: Investigation; Validation; Writing – review & editing. **Gerd Schulte‐Körne**: Supervision; Validation; Writing – review & editing. **Christian Wachinger**: Conceptualization; Data curation; Funding acquisition; Investigation; Supervision; Writing – review & editing.

## CONFLICT OF INTEREST STATEMENT

Sebastian Pölsterl is a full‐time employee of AstraZeneca. Richard Gaus is a part‐time employee of QuantCo. The remaining authors have declared that they have no competing or potential conflicts of interest.

### OPEN RESEARCH BADGES

This article has earned an Open Data badge for making publicly available the digitally‐shareable data necessary to reproduce the reported results. The data is available at https://nda.nih.gov/edit_collection.html?id=3104, http://dx.doi.org/10.15154/1503213, http://dx.doi.org/10.15154/1503306, http://dx.doi.org/10.15154/1503307, Source code: https://doi.org/10.5281/zenodo.7968586.

## ETHICAL CONSIDERATIONS

This study was designed and conducted in accordance with the ethical guidelines and principles of the Declaration of Helsinki. All procedures performed in this study involving human participants were in accordance with the ethical standards of the institutional research committee. The study used data from the ABCD study, which has received ethical approval and informed consent from all participants. All data were anonymized to protect participant confidentiality and privacy. No personal identifying information is included in any publication or presentation of the study result.

## Supporting information

Supporting Information S1

## Data Availability

Data from the ABCD Neurocognition Prediction Challenge are available online (https://nda.nih.gov/edit_collection.html?id=3104, http://dx.doi.org/10.15154/1503213, http://dx.doi.org/10.15154/1503306, http://dx.doi.org/10.15154/1503307). The code for performing all experiments is available at https://github.com/ai-med/abcd_study.

## References

[jcv212184-bib-0001] Alfaro‐Almagro, F. , McCarthy, P. , Afyouni, S. , Andersson, J. L. R. , Bastiani, M. , Miller, K. L. , Nichols, T. E. , & Smith, S. M. (2021). Confound modelling in UK Biobank brain imaging. NeuroImage, 224, 117002. 10.1016/j.neuroimage.2020.117002 32502668 PMC7610719

[jcv212184-bib-0002] Ambrosini, P. J. (2000). Historical development and present status of the schedule for affective disorders and schizophrenia for school‐age children (K‐SADS). Journal of the American Academy of Child & Adolescent Psychiatry, 39(1), 49–58. 10.1097/00004583-200001000-00016 10638067

[jcv212184-bib-0003] American Psychiatric Association . (2013). Diagnostic and statistical manual of mental disorders: DSM‐5 (5th ed.). American Psychiatric Association. 10.1176/appi.books.9780890425596

[jcv212184-bib-0004] Arbabshirani, M. R. , Plis, S. , Sui, J. , & Calhoun, V. D. (2017). Single subject prediction of brain disorders in neuroimaging: Promises and pitfalls. NeuroImage, 145, 137–165. 10.1016/j.neuroimage.2016.02.079 27012503 PMC5031516

[jcv212184-bib-0005] Astle, D. E. , Holmes, J. , Kievit, R. , & Gathercole, S. E. (2022). Annual Research Review: The transdiagnostic revolution in neurodevelopmental disorders. Journal of Child Psychology and Psychiatry, 63, (4). 397–417. 10.1111/jcpp.13481 34296774

[jcv212184-bib-0006] Barch, D. M. , Albaugh, M. D. , Avenevoli, S. , Chang, L. , Clark, D. B. , Glantz, M. D. , Hudziak, J. J. , Jernigan, T. L. , Tapert, S. F. , Alia‐Klein, N. , Potter, A. S. , Paulus, M. P. , Prouty, D. , Zucker, R. A. , Sher, K. J. , & Yurgelun‐Todd, D. (2018). Demographic, physical and mental health assessments in the adolescent brain and cognitive development study: Rationale and description. Developmental Cognitive Neuroscience, 32, 55–66. 10.1016/j.dcn.2017.10.010 29113758 PMC5934320

[jcv212184-bib-0007] Barch, D. M. , Albaugh, M. D. , Baskin‐Sommers, A. , Bryant, B. E. , Clark, D. B. , Dick, A. S. , Feczko, E. , Foxe, J. J. , Gee, D. G. , Giedd, J. , Glantz, M. D. , Hudziak, J. J. , Karcher, N. R. , LeBlanc, K. , Maddox, M. , McGlade, E. C. , Mulford, C. , Nagel, B. J. , Neigh, G. , …, & Xie, L. (2021). Demographic and mental health assessments in the adolescent brain and cognitive development study: Updates and age‐related trajectories. Developmental Cognitive Neuroscience, 52, 101031. 10.1016/j.dcn.2021.101031 34742018 PMC8579129

[jcv212184-bib-0008] Beauchaine, T. P. , & Hinshaw, S. P. (2020). RDoC and psychopathology among youth: Misplaced assumptions and an agenda for future research. Journal of Clinical Child and Adolescent Psychology, 49(3), 322–340. 10.1080/15374416.2020.1750022 32525746 PMC7495028

[jcv212184-bib-0009] Bird, H. R. , Gould, M. S. , & Staghezza, B. (1992). Aggregating data from multiple informants in child psychiatry epidemiological research. Journal of the American Academy of Child & Adolescent Psychiatry, 31(1), 78–85. 10.1097/00004583-199201000-00012 1537785

[jcv212184-bib-0010] Borsboom, D. , Cramer, A. O. J. , & Kalis, A. (2019). Brain disorders? Not really: Why network structures block reductionism in psychopathology research. Behavioral and Brain Sciences, 42, e2. 10.1017/S0140525X17002266 29361992

[jcv212184-bib-0011] Brodersen, K. H. , Ong, C. S. , Stephan, K. E. , & Buhmann, J. M. (2010). The balanced accuracy and its posterior distribution. In 20th international conference on pattern recognition (pp. 3121–3124). IEEE. 10.1109/ICPR.2010.764

[jcv212184-bib-0012] Bzdok, D. , & Meyer‐Lindenberg, A. (2018). Machine learning for precision psychiatry: Opportunities and challenges. Biological Psychiatry: Cognitive Neuroscience and Neuroimaging, 3(3), 223–230. 10.1016/j.bpsc.2017.11.007 29486863

[jcv212184-bib-0013] Casey, B. J. , Cannonier, T. , Conley, M. I. , Cohen, A. O. , Barch, D. M. , Heitzeg, M. M. , Soules, M. E. , Teslovich, T. , Dellarco, D. V. , Orr, C. A. , Wager, T. D. , Banich, M. T. , Speer, N. K. , Sutherland, M. T. , Riedel, M. C. , Dick, A. S. , Bjork, J. M. , Thomas, K. M. , & Garavan, H. (2018). The adolescent brain cognitive development (ABCD) study: Imaging acquisition across 21 sites. Developmental Cognitive Neuroscience, 32, 43–54. 10.1016/j.dcn.2018.03.001 29567376 PMC5999559

[jcv212184-bib-0014] Compton, W. M. , Dowling, G. J. , & Garavan, H. (2019). Ensuring the best use of data: The adolescent brain cognitive development study. JAMA Pediatrics, 173(9), 809–810. 10.1001/jamapediatrics.2019.2081 31305867 PMC8056387

[jcv212184-bib-0015] De Los Reyes, A. , Augenstein, T. M. , Wang, M. , Thomas, S. A. , Drabick, D. A. G. , Burgers, D. E. , & Rabinowitz, J. (2015). The validity of the multi‐informant approach to assessing child and adolescent mental health. Psychological Bulletin, 141(4), 858–900. 10.1037/a0038498 25915035 PMC4486608

[jcv212184-bib-0016] First, M. B. , Drevets, W. C. , Carter, C. , Dickstein, D. P. , Kasoff, L. , Kim, K. L. , McConathy, J. , Rauch, S. , Saad, Z. S. , Savitz, J. , Seymour, K. E. , Sheline, Y. I. , & Zubieta, J. K. (2018). Clinical applications of neuroimaging in psychiatric disorders. American Journal of Psychiatry, 175(9), 915–916. 10.1176/appi.ajp.2018.1750701 30173550 PMC6583905

[jcv212184-bib-0017] B. Fischl (2012). FreeSurfer. In: NeuroImage, 62.2(2), 774–781. 10.1016/j.neuroimage.2012.01.021 PMC3685476 22248573

[jcv212184-bib-0018] Friedman, J. H. (2001). Greedy function approximation: A gradient boosting machine. Annals of Statistics, 29(5), 1189–1232. 10.2307/2699986

[jcv212184-bib-0019] Garavan, H. , Bartsch, H. , Conway, K. , Decastro, A. , Goldstein, R. Z. , Heeringa, S. , Jernigan, T. , Potter, A. , Thompson, W. , & Zahs, D. (2018). Recruiting the ABCD sample: Design considerations and procedures. Developmental Cognitive Neuroscience, 32, 16–22. 10.1016/j.dcn.2018.04.004 29703560 PMC6314286

[jcv212184-bib-0020] Goodkind, M. , Eickhoff, S. B. , Oathes, D. J. , Jiang, Y. , Chang, A. , Jones‐Hagata, L. B. , Ortega, B. N. , Zaiko, Y. V. , Roach, E. L. , Grieve, S. M. , Galatzer‐Levy, I. , Fox, P. T. , Etkin, A. , & Korgaonkar, M. S. (2015). Identification of a common neurobiological substrate for mental illness. JAMA Psychiatry, 72(4), 305–315. 10.1001/jamapsychiatry.2014.2206 25651064 PMC4791058

[jcv212184-bib-0021] Hanley, J. A. , & McNeil, B. J. (1982). The meaning and use of the area under a receiver operating characteristic (ROC) curve. Radiology, 143(1), 29–36. http://www.ncbi.nlm.nih.gov/pubmed/7063747 7063747 10.1148/radiology.143.1.7063747

[jcv212184-bib-0022] Heeringa, S. G. , & Berglund, P. A. (2020). A guide for population‐based analysis of the Adolescent Brain Cognitive Development (ABCD) Study baseline data. bioRxiv. 10.1101/2020.02.10.942011

[jcv212184-bib-0023] Jollans, L. , & Whelan, R. (2018). Neuromarkers for mental disorders: Harnessing population neuroscience. Frontiers in Psychiatry, 9, 242. https://www.ncbi.nlm.nih.gov/pmc/articles/PMC5998767/pdf/fpsyt-09-00242.pdf 29928237 10.3389/fpsyt.2018.00242 PMC5998767

[jcv212184-bib-0024] Kambeitz, J. , Cabral, C. , Sacchet, M. D. , Gotlib, I. H. , Zahn, R. , Serpa, M. H. , Walter, M. , Falkai, P. , & Koutsouleris, N. (2017). Detecting neuroimaging biomarkers for depression: A meta‐analysis of multivariate pattern recognition studies. Biological Psychiatry, 82(5), 330–338. 10.1016/j.biopsych.2016.10.028 28110823 PMC11927514

[jcv212184-bib-0025] Kapur, S. , Phillips, A. G. , & Insel, T. R. (2012). Why has it taken so long for biological psychiatry to develop clinical tests and what to do about it? Molecular Psychiatry, 17(12), 1174–1179. 10.1038/mp.2012.105 22869033

[jcv212184-bib-0026] Karcher, N. R. , & Barch, D. M. (2021). The ABCD study: Understanding the development of risk for mental and physical health outcomes. Neuropsychopharmacology, 46(1), 131–142. 10.1038/s41386-020-0736-6 32541809 PMC7304245

[jcv212184-bib-0027] Kaufman, J. , Birmaher, B. , Brent, D. , Rao, U. M. A. , Flynn, C. , Moreci, P. , Williamson, D. , & Ryan, N. (1997). Schedule for affective disorders and schizophrenia for school‐age children‐present and lifetime version (K‐SADS‐PL): Initial reliability and validity data. Journal of the American Academy of Child & Adolescent Psychiatry, 36(7), 980–988. 10.1097/00004583-199707000-00021 9204677

[jcv212184-bib-0028] Kennedy, J. T. , Harms, M. P. , Korucuoglu, O. , Astafiev, S. V. , Barch, D. M. , Thompson, W. K. , Bjork, J. M. , & Anokhin, A. P. (2022). Reliability and stability challenges in ABCD task fMRI data. NeuroImage, 252, 119046. 10.1016/j.neuroimage.2022.119046 35245674 PMC9017319

[jcv212184-bib-0029] Kirkpatrick, R. H. , Munoz, D. P. , Khalid‐Khan, S. , & Booij, L. (2020). Methodological and clinical challenges associated with biomarkers for psychiatric disease: A scoping review. Journal of Psychiatric Research, 143, 572–579. 10.1016/j.jpsychires.2020.11.023 33221025

[jcv212184-bib-0030] Lahey, B. B. , Applegate, B. , Barkley, R. A. , Garfinkel, B. , McBurnett, K. , Kerdyk, L. , Greenhill, L. , Hynd, G. W. , Frick, P. J. , & Newcorn, J. (1994). DSM‐IV field trials for oppositional defiant disorder and conduct disorder in children and adolescents. American Journal of Psychiatry, 151(8), 1163–1171. 8037251 10.1176/ajp.151.8.1163

[jcv212184-bib-0031] Love, S. , Perry, A. , Ironside, J. , & Budka, H. (2018). Greenfield’s neuropathology‐two volume set. CRC Press.

[jcv212184-bib-0032] Martel, M. M. , Markon, K. , & Smith, G. T. (2017). Research Review: Multi‐informant integration in child and adolescent psychopathology diagnosis. Journal of Child Psychology and Psychiatry, 58(2), 116–128. 10.1111/jcpp.12611 27492280 PMC5247337

[jcv212184-bib-0033] Newman, D. L. , Moffitt, T. E. , Caspi, A. , & Silva, P. A. (1998). Comorbid mental disorders: Implications for treatment and sample selection. Journal of Abnormal Psychology, 107(2), 305–311. 10.1037/0021-843x.107.2.305 9604559

[jcv212184-bib-0034] Nielsen, A. N. , Barch, D. M. , Petersen, S. E. , Schlaggar, B. L. , & Greene, D. J. (2020). Machine learning with neuroimaging: Evaluating its applications in psychiatry. Biological Psychiatry: Cognitive Neuroscience and Neuroimaging, 5(8), 791–798. 10.1016/j.bpsc.2019.11.007 31982357 PMC8746222

[jcv212184-bib-0035] Ojala, M. , & Garriga, G. C. (2010). Permutation tests for studying classifier performance. Journal of Machine Learning Research, 11, 1833–1863. 10.1109/ICDM.2009.108

[jcv212184-bib-0036] Owens, M. M. , Allgaier, N. , Hahn, S. , Yuan, D. , Albaugh, M. , Adise, S. , Chaarani, B. , Ortigara, J. , Juliano, A. , Potter, A. , & Garavan, H. (2021). Multimethod investigation of the neurobiological basis of ADHD symptomatology in children aged 9‐10: Baseline data from the ABCD study. Translational Psychiatry, 11(1), 64. 10.1038/s41398-020-01192-8 33462190 PMC7813832

[jcv212184-bib-0037] Piacentini, J. C. , Cohen, P. , & Cohen, J. (1992). Combining discrepant diagnostic information from multiple sources: Are complex algorithms better than simple ones? Journal of Abnormal Child Psychology, 20(1), 51–63. 10.1007/bf00927116 1548394

[jcv212184-bib-0038] Pohl, K. M. , Thompson, W. K. , Adeli, E. , Landman, B. A. , Linguraru, M. G. , & Tapert, S. F. (2021). Adolescent brain cognitive development neurocognitive prediction challenge. Retrieved May 11, 2021, from https://sibis.sri.com/abcd-np-challenge/

[jcv212184-bib-0039] Pohl, K. M. , Thompson, W. K. , Adeli, E. , & Linguraru, M. G. (2019). Adolescent brain cognitive development neurocognitive prediction: First challenge, ABCD‐NP 2019, held in conjunction with MICCAI 2019 (Vol. 11791). Springer Nature. October 13, 2019, Proceedings.

[jcv212184-bib-0040] Rashid, B. , & Calhoun, V. (2020). Towards a brain‐based predictome of mental illness. Human Brain Mapping, 41(12), 3468–3535. 10.1002/hbm.25013 32374075 PMC7375108

[jcv212184-bib-0041] Read, J. , Pfahringer, B. , Holmes, G. , & Frank, E. (2011). Classifier chains for multi‐label classification. Machine Learning, 85(3), 333–359. 10.1007/s10994-011-5256-5

[jcv212184-bib-0042] Reiss, F. (2013). Socioeconomic inequalities and mental health problems in children and adolescents: A systematic review. Social Science & Medicine, 90, 24–31. 10.1016/j.socscimed.2013.04.026 23746605

[jcv212184-bib-0043] Rohlfing, T. , Zahr, N. M. , Sullivan, E. V. , & Pfefferbaum, A. (2010). The SRI24 multichannel atlas of normal adult human brain structure. Human Brain Mapping, 31(5), 798–819. 10.1002/hbm.20906 20017133 PMC2915788

[jcv212184-bib-0044] Sakai, K. , & Yamada, K. (2019). Machine learning studies on major brain diseases: 5‐Year trends of 2014–2018. Japanese Journal of Radiology, 37(1), 34–72. 10.1007/s11604-018-0794-4 30498877

[jcv212184-bib-0045] Scheinost, D. , Noble, S. , Horien, C. , Greene, A. S. , Lake, E. M. , Salehi, M. , Gao, S. , Shen, X. , O'Connor, D. , Barron, D. S. , Yip, S. W. , Rosenberg, M. D. , & Constable, R. T. (2019). Ten simple rules for predictive modeling of individual differences in neuroimaging. NeuroImage, 193, 35–45. 10.1016/j.neuroimage.2019.02.057 30831310 PMC6521850

[jcv212184-bib-0046] Schnack, H. G. (2019). Improving individual predictions: Machine learning approaches for detecting and attacking heterogeneity in schizophrenia (and other psychiatric diseases). Schizophrenia Research, 214, 34–42. 10.1016/j.schres.2017.10.023 29074332

[jcv212184-bib-0047] Schnack, H. G. , & Kahn, R. S. (2016). Detecting neuroimaging biomarkers for psychiatric disorders: Sample size matters. Frontiers in Psychiatry, 7, 50. 10.3389/fpsyt.2016.00050 27064972 PMC4814515

[jcv212184-bib-0048] Snoek, J. , Larochelle, H. , & Adams, R. P. (2012). Practical Bayesian optimization of machine learning algorithms.

[jcv212184-bib-0049] Snoek, L. , Miletić, S. , & Scholte, H. S. (2019). How to control for confounds in decoding analyses of neuroimaging data. NeuroImage, 184, 741–760. 10.1016/j.neuroimage.2018.09.074 30268846

[jcv212184-bib-0050] Sui, J. , Jiang, R. , Bustillo, J. , & Calhoun, V. (2020). Neuroimaging‐based individualized prediction of cognition and behavior for mental disorders and health: Methods and promises. Biological Psychiatry, 88(11), 818–828. 10.1016/j.biopsych.2020.02.016 32336400 PMC7483317

[jcv212184-bib-0051] Tamnes, C. K. , Herting, M. M. , Goddings, A.‐L. , Meuwese, R. , Blakemore, S.‐J. , Dahl, R. E. , Güroğlu, B. , Raznahan, A. , Sowell, E. R. , Mills, K. L. , & Crone, E. A. (2017). Development of the cerebral cortex across adolescence: A multisample study of inter‐related longitudinal changes in cortical volume, surface area, and thickness. Journal of Neuroscience, 37(12), 3402–3412. 10.1523/jneurosci.3302-16.2017 28242797 PMC5373125

[jcv212184-bib-0052] Thompson, W. K. , Barch, D. M. , Bjork, J. M. , Gonzalez, R. , Nagel, B. J. , Nixon, S. J. , & Luciana, M. (2019). The structure of cognition in 9 and 10 year‐old children and associations with problem behaviors: Findings from the ABCD study’s baseline neurocognitive battery. Developmental Cognitive Neuroscience, 36, 100606. 10.1016/j.dcn.2018.12.004 30595399 PMC6676481

[jcv212184-bib-0053] Townsend, L. , Kobak, K. , Kearney, C. , Milham, M. , Andreotti, C. , Escalera, J. , Alexander, L. , Gill, M. K. , Birmaher, B. , Rice, D. , Deep, A. , Kaufman, J. , & Sylvester, R. (2020). Development of three web‐based computerized versions of the Kiddie schedule for affective disorders and schizophrenia child psychiatric diagnostic interview: Preliminary validity data. Journal of the American Academy of Child & Adolescent Psychiatry, 59(2), 309–325. 10.1016/j.jaac.2019.05.009 31108163

[jcv212184-bib-0054] van Erp, T. G. M. , Walton, E. , Hibar, D. P. , Schmaal, L. , Jiang, W. , Glahn, D. C. , Pearlson, G. D. , Yao, N. , Fukunaga, M. , Hashimoto, R. , Okada, N. , Yamamori, H. , Bustillo, J. R. , Clark, V. P. , Agartz, I. , Mueller, B. A. , Cahn, W. , de Zwarte, S. M. , Hulshoff Pol, H. E. , …, & Orhan, F. (2018). Cortical brain abnormalities in 4474 individuals with schizophrenia and 5098 control subjects via the enhancing neuro imaging genetics through meta analysis (ENIGMA) consortium. Biological Psychiatry, 84(9), 644–654. 10.1016/j.biopsych.2018.04.023 29960671 PMC6177304

[jcv212184-bib-0055] Wachinger, C. , Rieckmann, A. , & Pölsterl, S. (2020). Detect and correct bias in multi‐site neuroimaging datasets. Medical Image Analysis, 67, 101879. 10.1016/j.media.2020.101879 33152602

[jcv212184-bib-0056] Wolfers, T. , Buitelaar, J. K. , Beckmann, C. F. , Franke, B. , & Marquand, A. F. (2015). From estimating activation locality to predicting disorder: A review of pattern recognition for neuroimaging‐based psychiatric diagnostics. Neuroscience & Biobehavioral Reviews, 57, 328–349. 10.1016/j.neubiorev.2015.08.001 26254595

[jcv212184-bib-0057] Woo, C.‐W. , Chang, L. J. , Lindquist, M. A. , & Wager, T. D. (2017). Building better biomarkers: Brain models in translational neuroimaging. Nature Neuroscience, 20(3), 365–377. 10.1038/nn.4478 28230847 PMC5988350

[jcv212184-bib-0058] Wu, M.‐J. , Wu, H. E. , Mwangi, B. , Sanches, M. , Selvaraj, S. , Zunta‐Soares, G. B. , & Soares, J. C. (2015). Prediction of pediatric unipolar depression using multiple neuromorphometric measurements: A pattern classification approach. Journal of Psychiatric Research, 62, 84–91. 10.1016/j.jpsychires.2015.01.015 25687738 PMC4355046

[jcv212184-bib-0059] Youden, W. (1950). Index for rating diagnostic tests. Cancer, 3(1), 32–35. 10.1002/1097-0142(1950)3:1<32::aid-cncr2820030106>3.0.co;2-3 15405679

[jcv212184-bib-0060] Zhang, C. , Liu, C. , Zhang, X. , & Almpanidis, G. (2017). An up‐to‐date comparison of state‐of‐the‐art classification algorithms. Expert Systems with Applications, 82, 128–150. 10.1016/j.eswa.2017.04.003

[jcv212184-bib-0061] Zhang‐James, Y. , Helminen, E. C. , Liu, J. , Group, E.‐A. W. , Franke, B. , Hoogman, M. , Faraone, S. V. , Gabel, M. C. , Harrison, N. A. , Lazaro, L. , Lera‐Miguel, S. , Louza, M. R. , Nicolau, R. , Rosa, P. G. P. , Schulte‐Rutte, M. , Zanetti, M. V. , Ambrosino, S. , Asherson, P. , & Banaschewski, T. (2021). Evidence for similar structural brain anomalies in youth and adult attention‐deficit/hyperactivity disorder: A machine learning analysis. Translational Psychiatry, 11(1), 82. 10.1038/s41398-021-01201-4 33526765 PMC7851168

