# Supplementary material for: Can we diagnose mental disorders in children? A large‐scale assessment of machine learning on structural neuroimaging of 6916 children in the adolescent brain cognitive development study
Source: JCPP Adv. 2023 Jun 28;3(4):e12184. doi: 10.1002/jcv2.12184 (PMC10694548; doi:10.1002/jcv2.12184)
Supplement: Supplementary file 1 — Supporting Information S1 [file JCV2-3-e12184-s001.docx]

# ­­­­Supporting Information

# Appendix S1

## Imaging data processing

We obtained the 136 FreeSurfer features from T1-weighted sMRI data using the procedure described in Pölsterl et al. (Pölsterl, Gutiérrez-Becker, Sarasua, Guha Roy, & Wachinger, 2019). First, we used the FreeSurfer (Fischl, 2012) software (v 5.3) to automatically segment T1-weighted sMRI images. Second, we applied the FreeSurfer scripts asegstats2table and aparcstats2table to the segmentations, producing 136 measurements of regional volumes, cortical thicknesses, and surface hole numbers. A full list of these FreeSurfer features is given in Table S2.

## Residualizing Measurements

To account for known confounding effects we residualized measurements with respect to the sociodemographic variables age, sex, marriage status of parents, study site, highest parental education, ethnicity, and supratentorial brain volume using the approach proposed by Snoek et al. (L. Snoek, Miletić, & Scholte, 2019). We fitted ordinary least squares models using statsmodels v0.12.0 (Seabold & Perktold, 2010) on the data, estimating each brain structural feature from the sociodemographic variables, and then obtained the residuals of the created models. Lastly, we removed all features with a variance less than 0.001 (four features, or 1.55% of all features, were removed in this process) and rescaled the remaining ones by subtracting the median and dividing by the range between the 5th and 95th percentiles as done previously in our submission to the ABCD Neurocognitive Prediction Challenge (Pölsterl et al., 2019). To prevent information leakage from the validation and test sets into the training set, all fitting operations were performed only on the training set prior to transforming the training, validation and test sets.

## Machine learning models

### Classifier chain ensemble of gradient boosting models (GBM CCE)

Ensembles of gradient boosted trees (GBMs; (Friedman, 2001)) are powerful machine learning models that consist of a set of decision trees where each tree predicts the gradient of the loss of the previous tree’s prediction. We used GBMs with 1000 trees and to increase robustness, each tree was trained on a random 50% stratified subsample of the total training set. Given that a large number of uninformative features can degrade model performance, we also applied automated feature selection via elastic net regularization (Zou & Hastie, 2005). Both regularization strength parameters and the step size shrinkage were tuned with Bayesian hyperparameter optimization (J. Snoek, Larochelle, & Adams, 2012) so that the performance of the model on the validation set, after being fit to the training set, was optimized. The sampling priors of these hyperparameters are given in Table S4. We used an iteration limit of 30 on Bayesian optimization. During each iteration, the validation error was calculated as the mean plus standard deviation of the binary cross entropy errors on 100 random 66.6% stratified subsamples of the validation set. The GBM was implemented using the XGBoost Python library (Chen & Guestrin, 2016).

Classifier chains (Read, Pfahringer, Holmes, & Frank, 2011) effectively exploit interdependencies among labels by chaining a number of individual base classifiers so that the predictions of a previous one are used as additional features for the next. We constructed such classifier chains with the hyperparameter-optimized GBM as a base classifier. To further increase robustness of predictions, we created an ensemble of ten such classifier chains (classifier chain ensemble, CCE), each with a different random order of label predictions. The final prediction was calculated as the mean of all ten classifier chains’ predictions.

### Logistic regression classifier

A simple logistic regression classifier (LRC) was used as a linear benchmark model for comparing the performance of the more complex GBM CCE. For each response separately, a logistic regression model with fixed hyperparameters was fit to the concatenated training and validation sets and evaluated on the test set. We used L2 regularization with inverse regularization strength C = 1.0, balanced class weights, L-BFGS optimizer, and a fixed maximum number of iterations of 300. We used the implementation in the scikit-learn library (Pedregosa et al., 2011).

### Implementation

The GBM was implemented in the Python XGBoost library (v1.3.3) (Chen & Guestrin, 2016), the LRC and classifier chain ensemble in the scikit-learn library (v0.23.2) (Pedregosa et al., 2011), and hyper-parameter optimization in scikit-optimize (v0.8.1) (Head, Kumar, Nahrstaedt, Louppe, & Shcherbatyi, 2020).

## Permutation-test for Assessing Classifier Performance

To assess whether classifiers detected true patterns in the data, we evaluated the performance of all models using the permutation test described in (Ojala & Garriga, 2010). We created 500 random permutations of the original dataset by randomly reassigning the sets of disorder labels among all subjects – while preserving the relative combinations of disorders within cases. On each of these permuted datasets, we performed a single 5-fold cross-validation, as described above, which yielded 5 different test set AUROC values which were averaged to obtain $\bar{AUROC}_{perm,i}$, where $i=1,\ldots,500$ denotes the index of the permuted dataset. Lastly, we computed a p-value for each AUROC based on the permutation test described in (Ojala & Garriga, 2010). The initial significance level was chosen at $\alpha=0.05$. As we performed 20 independent permutation tests (one for each of the models GBM-CCE and LRC and each of the eight disorders), we applied Bonferroni correction for multiple comparisons, resulting in an adjusted significance level $\alpha_{adj}=\frac{0.05}{20}=0.0025$.

To estimate the effect of adjusting for sociodemographic variables via residualization, we obtained an additional AUROC value based on data without this adjustment, using the same 30-times repeated 5-fold cross-validation scheme as described above. All plots were generated with the Python libraries matplotlib (Hunter, 2007) and seaborn (Waskom, 2021).

# References

Chen, T., & Guestrin, C. (2016). XGBoost. In Proceedings of the 22nd ACM SIGKDD International Conference on Knowledge Discovery and Data Mining (pp. 785–794). New York, NY, USA: ACM. https://doi.org/10.1145/2939672.2939785

Fischl, B. (2012). FreeSurfer. NeuroImage, 62(2), 774–781.

Friedman, J. H. (2001). Greedy Function Approximation: A Gradient Boosting Machine. The Annals of Statistics, 29(5), 1189–1232. https://doi.org/10.2307/2699986

Head, T., Kumar, M., Nahrstaedt, H., Louppe, G., & Shcherbatyi, I. (2020). scikit-optimize/scikit-optimize. https://doi.org/10.5281/ZENODO.4014775

Hunter, J. D. (2007). Matplotlib: A 2D graphics environment. IEEE Annals of the History of Computing, 9(03), 90–95.

Ojala, M., & Garriga, G. C. (2010). Permutation Tests for Studying Classifier Performance. Journal of Machine Learning Research, 11, 1833–1863. https://doi.org/10.1109/ICDM.2009.108

Pedregosa, F., Varoquaux, G., Gramfort, A., Michel, V., Thirion, B., Grisel, O., … Dubourg, V. (2011). Scikit-learn: Machine learning in Python. The Journal of Machine Learning Research, 12, 2825–2830.

Pölsterl, S., Gutiérrez-Becker, B., Sarasua, I., Guha Roy, A., & Wachinger, C. (2019). Prediction of Fluid Intelligence from T1-Weighted Magnetic Resonance Images. In K. M. Pohl, W. K. Thompson, E. Adeli, & M. G. Linguraru (Eds.), Adolescent Brain Cognitive Development Neurocognitive Prediction (pp. 35–46). Cham: Springer International Publishing.

Read, J., Pfahringer, B., Holmes, G., & Frank, E. (2011). Classifier chains for multi-label classification. Machine Learning, 85(3), 333–359. https://doi.org/10.1007/s10994-011-5256-5

Seabold, S., & Perktold, J. (2010). Statsmodels: Econometric and statistical modeling with python. In 9th Python in Science Conference.

Snoek, J., Larochelle, H., & Adams, R. P. (2012). Practical Bayesian Optimization of Machine Learning Algorithms. https://doi.org/1206.2944

Snoek, L., Miletić, S., & Scholte, H. S. (2019). How to control for confounds in decoding analyses of neuroimaging data. NeuroImage, 184, 741–760.

Waskom, M. L. (2021). Seaborn: statistical data visualization. Journal of Open Source Software, 6(60), 3021.

Zou, H., & Hastie, T. (2005). Regularization and variable selection via the elastic net. Journal of the Royal Statistical Society: Series B Statistical Methodology, 67(2), 301–320.

# ­­­­Appendix S2. Analysis based on SRI24 features

## Imaging data processing

The full set of 122 SRI24 features was obtained from the National Institute of Mental Health Data Archive^[[1]](#footnote-2)^. Originally, these features were derived from T1-weighted sMRI scans in the context of the ABCD Neurocognitive Prediction Challenge (Pohl, Thompson, Adeli, & Linguraru, 2019) and the full procedure is described on the website of the challenge (Pohl et al., 2021). In a first step, the raw T1-weighted sMRI data was converted into NIfTI4 files using the Minimal Processing Pipeline of the ABCD consortium (Hagler Jr et al., 2019). Then, the processing pipeline described in the data supplement of Pfefferbaum et al. (Pfefferbaum et al., 2018) was applied to the NIfTI files. This pipeline involved noise removal, repeated correction of field inhomogeneity, non-rigid alignment of the SRI24 atlas (Rohlfing, Zahr, Sullivan, & Pfefferbaum, 2010) to T1-weighted sMRI, refinement of the brain mask by majority voting over maps extracted by different softwares, and segmentation of the brain tissue into gray matter, white matter, and cerebrospinal fluid. Finally, the gray matter tissue was further parcellated according to the SRI24 atlas. For quality control, the skull-stripped T1-weighted images and corresponding segmentations were affinely mapped to the SRI24 atlas and visually inspected. Segmentations that failed a two-tier quality check were rejected from the data set (Pohl et al., 2021). A full list of the resulting SRI24 features is given in Table S3.

## Residualizing Measurements

The SRI24 features were residualized in exactly the same way as the FreeSurfer features (Appendix S1) with respect to the sociodemographic variables age, sex, marriage status of parents, study site, highest parental education, ethnicity, and supratentorial brain volume.

## Results

### Predictive Performance

Figure S1 summarizes the predictive performance in terms of the AUROC for all ten disorders, and whether the model has found a real pattern in the data based on permutation testing.

Neither the classifier chain ensemble of gradient boosting models (GBM-CCE) nor the logistic regression classifier (LRC) achieved statistically significant predictions for any disorder. The diagnoses with the lowest p-values were attention deficit hyperactivity disorder (ADHD, p = 0.016 with GBM-CCE), oppositional defiant disorder (p = 0.020 with GBM-CCE, p = 0.026 with LRC), social anxiety disorder (p = 0.022 with LRC), and bipolar disorder (BD, p = 0.034 with LRC). While these predictions do not reach the significance level of $\alpha_{adj}=0.0025$, the associated diagnoses overlap with the set of diagnoses that could be predicted significantly based on the FreeSurfer features (ADHD and BD).

# References

Hagler Jr, D. J., Hatton, S., Cornejo, M. D., Makowski, C., Fair, D. A., Dick, A. S., … Harms, M. P. (2019). Image processing and analysis methods for the Adolescent Brain Cognitive Development Study. NeuroImage, 202, 116091.

Pfefferbaum, A., Kwon, D., Brumback, T., Thompson, W. K., Cummins, K., Tapert, S. F., … others. (2018). Altered Brain Developmental Trajectories in Adolescents After Initiating Drinking. American Journal of Psychiatry, 175(4), 370–380.

Pohl, K. M., Thompson, W. K., Adeli, E., Landman, B. A., Linguraru, M. G., & Tapert, S. F. (2021). Adolescent Brain Cognitive Development Neurocognitive Prediction Challenge. Retrieved May 11, 2021, from https://sibis.sri.com/abcd-np-challenge/

Pohl, K. M., Thompson, W. K., Adeli, E., & Linguraru, M. G. (2019). Adolescent Brain Cognitive Development Neurocognitive Prediction: First Challenge, ABCD-NP 2019, Held in Conjunction with MICCAI 2019, Shenzhen, China, October 13, 2019, Proceedings (Vol. 11791). Springer Nature.

Rohlfing, T., Zahr, N. M., Sullivan, E. V, & Pfefferbaum, A. (2010). The SRI24 multichannel atlas of normal adult human brain structure. Human Brain Mapping, 31(5), 798–819.

**Figure S1. Violin plots of cross-validation results for SRI24 features.** For each disorder and both classifiers, the distribution of AUROC under the null-hypothesis of “no real pattern has been discovered” (in gray) is contrasted with the AUROC value (diamond) on the original dataset. Dashed diamonds show AUROC values on unpermuted data with no adjustment by sociodemographic confounders (see Table S6 for a statistical comparison with original AUROC values). Dashed line at $\bar{AUROC}=0.5$ corresponds to a classifier with no discriminative ability. **GBM-CCE:** Gradient boosting model classifier chain ensemble. **LRC:** Logistic regression classifier.


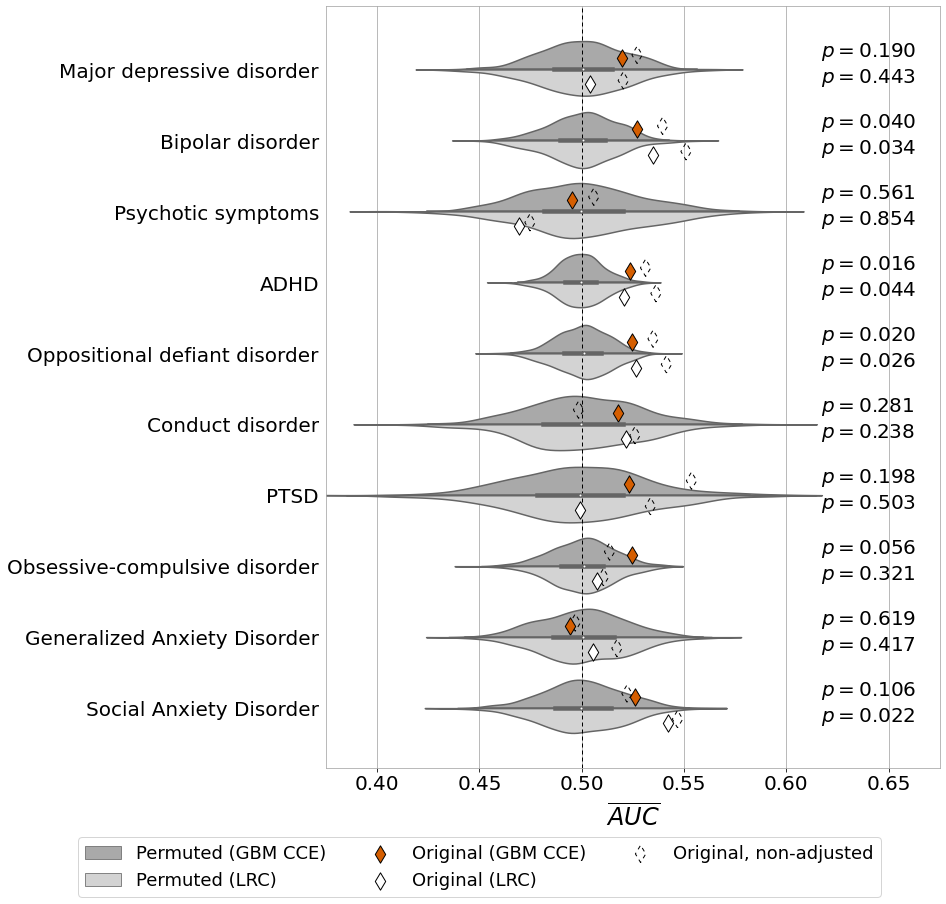


**Table S1**. Case numbers of all DSM-5 diagnoses that were considered for inclusion in this study (aggregated via OR rule) in the full ABCD baseline assessment (N = 11 875).

| Diagnosis | Number positives | Number negatives | Percent positive | Number unknowns |
| --- | --- | --- | --- | --- |
| Major Depressive Disorder | 569 | 11092 | 4.88 | 214 |
| Persistent Depressive Disorder (Dysthymia) | 24 | 11636 | 0.21 | 215 |
| Bipolar Disorder | 878 | 10790 | 7.53 | 207 |
| Psychotic Symptoms | 315 | 11406 | 2.69 | 154 |
| ADHD | 2192 | 9509 | 18.73 | 174 |
| Oppositional Defiant Disorder | 1667 | 10034 | 14.25 | 174 |
| Conduct Disorder | 374 | 11327 | 3.20 | 174 |
| PTSD | 231 | 11459 | 1.98 | 185 |
| Obsessive Compulsive Disorder | 1099 | 10591 | 9.40 | 185 |
| Generalized Anxiety Disorder | 579 | 11084 | 4.96 | 212 |
| Social Anxiety Disorder | 619 | 11043 | 5.31 | 213 |
| Panic Disorder | 32 | 11658 | 0.27 | 185 |
| Agoraphobia | 55 | 6217 | 0.88 | 5603 |
| Separation Anxiety Disorder | 1049 | 10661 | 8.96 | 165 |
| Binge-Eating Disorder | 88 | 0 | 100.00 | 11787 |
| Anorexia Nervosa | 6 | 0 | 100.00 | 11869 |
| Bulimia Nervosa | 7 | 0 | 100.00 | 11868 |
| Disruptive Mood Dysregulation Disorder | 22 | 5966 | 0.37 | 5887 |
| Substance Use Disorder | 1 | 0 | 100.00 | 11874 |

**Table S2**. Full list of FreeSurfer features used in this study.

| Feature type | Feature code | Feature description |
| --- | --- | --- |
| Cortical thickness | FS_lh_bankssts_thickness | Left hemisphere banks superior temporal sulcus thickness |
|  | FS_lh_caudalanteriorcingulate_thickness | Left hemisphere caudal anterior-cingulate cortex thickness |
|  | FS_lh_caudalmiddlefrontal_thickness | Left hemisphere caudal middle frontal gyrus thickness |
|  | FS_lh_cuneus_thickness | Left hemisphere cuneus cortex thickness |
|  | FS_lh_entorhinal_thickness | Left hemisphere entorhinal cortex thickness |
|  | FS_lh_fusiform_thickness | Left hemisphere fusiform gyrus thickness |
|  | FS_lh_inferiorparietal_thickness | Left hemisphere inferior parietal cortex thickness |
|  | FS_lh_inferiortemporal_thickness | Left hemisphere inferior temporal gyrus thickness |
|  | FS_lh_isthmuscingulate_thickness | Left hemisphere isthmus–cingulate cortex thickness |
|  | FS_lh_lateraloccipital_thickness | Left hemisphere lateral occipital cortex thickness |
|  | FS_lh_lateralorbitofrontal_thickness | Left hemisphere lateral orbital frontal cortex thickness |
|  | FS_lh_lingual_thickness | Left hemisphere lingual gyrus thickness |
|  | FS_lh_medialorbitofrontal_thickness | Left hemisphere medial orbital frontal cortex thickness |
|  | FS_lh_middletemporal_thickness | Left hemisphere middle temporal gyrus thickness |
|  | FS_lh_parahippocampal_thickness | Left hemisphere parahippocampal gyrus thickness |
|  | FS_lh_paracentral_thickness | Left hemisphere paracentral lobule thickness |
|  | FS_lh_parsopercularis_thickness | Left hemisphere pars opercularis thickness |
|  | FS_lh_parsorbitalis_thickness | Left hemisphere pars orbitalis thickness |
|  | FS_lh_parstriangularis_thickness | Left hemisphere pars triangularis thickness |
|  | FS_lh_pericalcarine_thickness | Left hemisphere pericalcarine cortex thickness |
|  | FS_lh_postcentral_thickness | Left hemisphere postcentral gyrus thickness |
|  | FS_lh_posteriorcingulate_thickness | Left hemisphere posterior-cingulate cortex thickness |
|  | FS_lh_precentral_thickness | Left hemisphere precentral gyrus thickness |
|  | FS_lh_precuneus_thickness | Left hemisphere precuneus cortex thickness |
|  | FS_lh_rostralanteriorcingulate_thickness | Left hemisphere rostral anterior cingulate cortex thickness |
|  | FS_lh_rostralmiddlefrontal_thickness | Left hemisphere rostral middle frontal gyrus thickness |
|  | FS_lh_superiorfrontal_thickness | Left hemisphere superior frontal gyrus thickness |
|  | FS_lh_superiorparietal_thickness | Left hemisphere superior parietal cortex thickness |
|  | FS_lh_superiortemporal_thickness | Left hemisphere superior temporal gyrus thickness |
|  | FS_lh_supramarginal_thickness | Left hemisphere supramarginal gyrus thickness |
|  | FS_lh_frontalpole_thickness | Left hemisphere frontal pole thickness |
|  | FS_lh_temporalpole_thickness | Left hemisphere temporal pole thickness |
|  | FS_lh_transversetemporal_thickness | Left hemisphere transverse temporal cortex thickness |
|  | FS_lh_insula_thickness | Left hemisphere insular cortex thickness |
|  | FS_lh_MeanThickness_thickness | Left hemisphere mean cortical thickness |
|  | FS_rh_bankssts_thickness | Right hemisphere banks superior temporal sulcus thickness |
|  | FS_rh_caudalanteriorcingulate_thickness | Right hemisphere caudal anterior-cingulate cortex thickness |
|  | FS_rh_caudalmiddlefrontal_thickness | Right hemisphere caudal middle frontal gyrus thickness |
|  | FS_rh_cuneus_thickness | Right hemisphere cuneus cortex thickness |
|  | FS_rh_entorhinal_thickness | Right hemisphere entorhinal cortex thickness |
|  | FS_rh_fusiform_thickness | Right hemisphere fusiform gyrus thickness |
|  | FS_rh_inferiorparietal_thickness | Right hemisphere inferior parietal cortex thickness |
|  | FS_rh_inferiortemporal_thickness | Right hemisphere inferior temporal gyrus thickness |
|  | FS_rh_isthmuscingulate_thickness | Right hemisphere isthmus–cingulate cortex thickness |
|  | FS_rh_lateraloccipital_thickness | Right hemisphere lateral occipital cortex thickness |
|  | FS_rh_lateralorbitofrontal_thickness | Right hemisphere lateral orbital frontal cortex thickness |
|  | FS_rh_lingual_thickness | Right hemisphere lingual gyrus thickness |
|  | FS_rh_medialorbitofrontal_thickness | Right hemisphere medial orbital frontal cortex thickness |
|  | FS_rh_middletemporal_thickness | Right hemisphere middle temporal gyrus thickness |
|  | FS_rh_parahippocampal_thickness | Right hemisphere parahippocampal gyrus thickness |
|  | FS_rh_paracentral_thickness | Right hemisphere paracentral lobule thickness |
|  | FS_rh_parsopercularis_thickness | Right hemisphere pars opercularis thickness |
|  | FS_rh_parsorbitalis_thickness | Right hemisphere pars orbitalis thickness |
|  | FS_rh_parstriangularis_thickness | Right hemisphere pars triangularis thickness |
|  | FS_rh_pericalcarine_thickness | Right hemisphere pericalcarine cortex thickness |
|  | FS_rh_postcentral_thickness | Right hemisphere postcentral gyrus thickness |
|  | FS_rh_posteriorcingulate_thickness | Right hemisphere posterior-cingulate cortex thickness |
|  | FS_rh_precentral_thickness | Right hemisphere precentral gyrus thickness |
|  | FS_rh_precuneus_thickness | Right hemisphere precuneus cortex thickness |
|  | FS_rh_rostralanteriorcingulate_thickness | Right hemisphere rostral anterior cingulate cortex thickness |
|  | FS_rh_rostralmiddlefrontal_thickness | Right hemisphere rostral middle frontal gyrus thickness |
|  | FS_rh_superiorfrontal_thickness | Right hemisphere superior frontal gyrus thickness |
|  | FS_rh_superiorparietal_thickness | Right hemisphere superior parietal cortex thickness |
|  | FS_rh_superiortemporal_thickness | Right hemisphere superior temporal gyrus thickness |
|  | FS_rh_supramarginal_thickness | Right hemisphere supramarginal gyrus thickness |
|  | FS_rh_frontalpole_thickness | Right hemisphere frontal pole thickness |
|  | FS_rh_temporalpole_thickness | Right hemisphere temporal pole thickness |
|  | FS_rh_transversetemporal_thickness | Right hemisphere transverse temporal cortex thickness |
|  | FS_rh_insula_thickness | Right hemisphere insular cortex thickness |
|  | FS_rh_MeanThickness_thickness | Right hemisphere mean cortical thickness |
| Regional volume | FS_Left-Lateral-Ventricle | Left lateral ventricle volume |
|  | FS_Left-Inf-Lat-Vent | Left inferior lateral ventricle volume |
|  | FS_Left-Cerebellum-White-Matter | Left cerebellum white matter volume |
|  | FS_Left-Cerebellum-Cortex | Left cerebellum cortex volume |
|  | FS_Left-Thalamus-Proper | Left thalamus volume |
|  | FS_Left-Caudate | Left caudate volume |
|  | FS_Left-Putamen | Left putamen volume |
|  | FS_Left-Pallidum | Left pallidum volume |
|  | FS_3rd-Ventricle | Third ventricle volume |
|  | FS_4th-Ventricle | Fourth ventricle volume |
|  | FS_Brain-Stem | Brain stem volume |
|  | FS_Left-Hippocampus | Left hippocampus volume |
|  | FS_Left-Amygdala | Left amygdala volume |
|  | FS_CSF | Cerebrospinal fluid volume |
|  | FS_Left-Accumbens-area | Left accumbens area volume |
|  | FS_Left-VentralDC | Left ventral diencephalon volume |
|  | FS_Left-vessel | Left vessel volume (non-specific) |
|  | FS_Left-choroid-plexus | Left choroid plexus volume |
|  | FS_Right-Lateral-Ventricle | Right lateral ventricle volume |
|  | FS_Right-Inf-Lat-Vent | Right inferior lateral ventricle volume |
|  | FS_Right-Cerebellum-White-Matter | Right cerebellum white matter volume |
|  | FS_Right-Cerebellum-Cortex | Right cerebellum cortex volume |
|  | FS_Right-Thalamus-Proper | Right thalamus volume |
|  | FS_Right-Caudate | Right caudate volume |
|  | FS_Right-Putamen | Right putamen volume |
|  | FS_Right-Pallidum | Right pallidum volume |
|  | FS_Right-Hippocampus | Right hippocampus volume |
|  | FS_Right-Amygdala | Right amygdala volume |
|  | FS_Right-Accumbens-area | Right accumbens area volume |
|  | FS_Right-VentralDC | Right ventral diencephalon volume |
|  | FS_Right-vessel | Right vessel volume (non-specific) |
|  | FS_Right-choroid-plexus | Right choroid plexus volume |
|  | FS_5th-Ventricle | Fifth ventricle volume |
|  | FS_WM-hypointensities | White matter hypointensities volume |
|  | FS_Left-WM-hypointensities | Left hemisphere white matter hypointensities volume |
|  | FS_Right-WM-hypointensities | Right hemisphere white matter hypointensities volume |
|  | FS_non-WM-hypointensities | Non-white matter hypointensities volume |
|  | FS_Left-non-WM-hypointensities | Left hemisphere non-white matter hypointensities volume |
|  | FS_Right-non-WM-hypointensities | Right hemisphere non-white matter hypointensities volume |
|  | FS_Optic-Chiasm | Optic chiasm volume |
|  | FS_CC_Posterior | Posterior corpus callosum volume |
|  | FS_CC_Mid_Posterior | Mid posterior corpus callosum volume |
|  | FS_CC_Central | Central corpus callosum volume |
|  | FS_CC_Mid_Anterior | Mid anterior corpus callosum volume |
|  | FS_CC_Anterior | Anterior corpus callosum volume |
|  | FS_BrainSegVol | Brain segmentation volume |
|  | FS_BrainSegVolNotVent | Brain segmentation volume without ventricles |
|  | FS_BrainSegVolNotVentSurf | Brain segmentation volume without ventricles from Surf |
|  | FS_lhCortexVol | Left hemisphere cortical gray matter volume |
|  | FS_rhCortexVol | Right hemisphere cortical gray matter volume |
|  | FS_CortexVol | Total cortical gray matter volume |
|  | FS_lhCorticalWhiteMatterVol | Left hemisphere cerebral white matter volume |
|  | FS_rhCorticalWhiteMatterVol | Right hemisphere cerebral white matter volume |
|  | FS_CorticalWhiteMatterVol | Total cerebral white matter volume |
|  | FS_SubCortGrayVol | Subcortical gray matter volume |
|  | FS_TotalGrayVol | Total gray matter volume |
|  | FS_SupraTentorialVol | Supratentorial volume |
|  | FS_SupraTentorialVolNotVent | Supratentorial volume without ventricles |
|  | FS_SupraTentorialVolNotVentVox | Supratentorial volume without ventricles voxel count |
|  | FS_MaskVol | Mask volume |
|  | FS_BrainSegVol-to-eTIV | Ratio of BrainSegVol to eTIV |
|  | FS_MaskVol-to-eTIV | Ratio fo MaskVol to eTIV |
|  | FS_EstimatedTotalIntraCranialVol | Estimated total intracranial volume (eTIV) |
| Number of surface holes | FS_lhSurfaceHoles | Number of defect holes in left hemisphere surfaces prior to fixing |
|  | FS_rhSurfaceHoles | Number of defect holes in right hemisphere surfaces prior to fixing |
|  | FS_SurfaceHoles | Total number of defect holes in surfaces prior to fixing |

**Table S3**. Full list of SRI24 features used in this study.

| Feature code | Feature description |
| --- | --- |
| sri24precentrallgm | Left precentral gyrus gray matter volume |
| sri24precentralrgm | Right precentral gyrus gray matter volume |
| sri24frontalsuplgm | Left superior frontal gyrus, dorsolater gray matter volume |
| sri24frontalsuprgm | Right superior frontal gyrus, dorsolater gray matter volume |
| sri24frontalsuporblgm | Left superior frontal gyrus, orbital part gray matter volume |
| sri24frontalsuporbrgm | Right superior frontal gyrus, orbital part gray matter volume |
| sri24frontalmidlgm | Left middle frontal gyrus, lateral part gray matter volume |
| sri24frontalmidrgm | Right middle frontal gyrus, lateral part gray matter volume |
| sri24frontalmidorblgm | Left middle frontal gyrus, orbital part gray matter volume |
| sri24frontalmidorbrgm | Right middle frontal gyrus, orbital part gray matter volume |
| sri24frontalinfoperlgm | Left opercular part of inferior frontal gyrus gray matter volume |
| sri24frontalinfoperrgm | Right opercular part of inferior frontal gyrus gray matter volume |
| sri24frontalinftrilgm | Left area triangularis gray matter volume |
| sri24frontalinftrirgm | Right area triangularis gray matter volume |
| sri24frontalinforblgm | Left orbital part of inferior frontal gyrus gray matter volume |
| sri24frontalinforbrgm | Right orbital part of inferior frontal gyrus gray matter volume |
| sri24rolandicoperlgm | Left rolandic operculum gray matter volume |
| sri24rolandicoperrgm | Right rolandic operculum gray matter volume |
| sri24suppmotorarealgm | Left supplementary motor area gray matter volume |
| sri24suppmotorareargm | Right supplementary motor area gray matter volume |
| sri24olfactorylgm | Left olfactory cortex gray matter volume |
| sri24olfactoryrgm | Right olfactory cortex gray matter volume |
| sri24frontalsupmediallgm | Left superior frontal gyrus, medial part gray matter volume |
| sri24frontalsupmedialrgm | Right superior frontal gyrus, medial part gray matter volume |
| sri24frontalmedorblgm | Left superior frontal gyrus, medial orbital part gray matter volume |
| sri24frontalmedorbrgm | Right superior frontal gyrus, medial orbital part gray matter volume |
| sri24rectuslgm | Left gyrus rectus gray matter volume |
| sri24rectusrgm | Right gyrus rectus gray matter volume |
| sri24insulalgm | Left insula gray matter volume |
| sri24insulargm | Right insula gray matter volume |
| sri24cingulumantlgm | Left anterior cingulate gyrus gray matter volume |
| sri24cingulumantrgm | Right anterior cingulate gyrus gray matter volume |
| sri24cingulummidlgm | Left middle cingulate gray matter volume |
| sri24cingulummidrgm | Right middle cingulate gray matter volume |
| sri24cingulumpostlgm | Left posterior cingulate gyrus gray matter volume |
| sri24cingulumpostrgm | Right posterior cingulate gyrus gray matter volume |
| sri24hippocampuslgm | Left hippocampus gray matter volume |
| sri24hippocampusrgm | Right hippocampus gray matter volume |
| sri24parahippocampallgm | Left parahippocampal gyrus gray matter volume |
| sri24parahippocampalrgm | Right parahippocampal gyrus gray matter volume |
| sri24amygdalalgm | Left amygdala gray matter volume |
| sri24amygdalargm | Right amygdala gray matter volume |
| sri24calcarinelgm | Left calcarine sulcus gray matter volume |
| sri24calcarinergm | Right calcarine sulcus gray matter volume |
| sri24cuneuslgm | Left cuneus gray matter volume |
| sri24cuneusrgm | Right cuneus gray matter volume |
| sri24linguallgm | Left lingual gyrus gray matter volume |
| sri24lingualrgm | Right lingual gyrus gray matter volume |
| sri24occipitalsuplgm | Left superior occipital gray matter volume |
| sri24occipitalsuprgm | Right superior occipital gray matter volume |
| sri24occipitalmidlgm | Left middle occipital gray matter volume |
| sri24occipitalmidrgm | Right middle occipital gray matter volume |
| sri24occipitalinflgm | Left inferior occipital gray matter volume |
| sri24occipitalinfrgm | Right inferior occipital gray matter volume |
| sri24fusiformlgm | Left fusiform gyrus gray matter volume |
| sri24fusiformrgm | Right fusiform gyrus gray matter volume |
| sri24postcentrallgm | Left postcentral gyrus gray matter volume |
| sri24postcentralrgm | Right postcentral gyrus gray matter volume |
| sri24parietalsuplgm | Left superior parietal lobule gray matter volume |
| sri24parietalsuprgm | Right superior parietal lobule gray matter volume |
| sri24parietalinflgm | Left inferior parietal lobule gray matter volume |
| sri24parietalinfrgm | Right inferior parietal lobule gray matter volume |
| sri24supramarginallgm | Left supramarginal gyrus gray matter volume |
| sri24supramarginalrgm | Right supramarginal gyrus gray matter volume |
| sri24angularlgm | Left angular gyrus gray matter volume |
| sri24angularrgm | Right angular gyrus gray matter volume |
| sri24precuneuslgm | Left precuneus gray matter volume |
| sri24precuneusrgm | Right precuneus gray matter volume |
| sri24paracentrallobulelgm | Left paracentral lobule gray matter volume |
| sri24paracentrallobulergm | Right paracentral lobule gray matter volume |
| sri24caudatelgm | Left caudate nucleus gray matter volume |
| sri24caudatergm | Right caudate nucleus gray matter volume |
| sri24putamenlgm | Left putamen gray matter volume |
| sri24putamenrgm | Right putamen gray matter volume |
| sri24pallidumlgm | Left globus pallidus gray matter volume |
| sri24pallidumrgm | Right globus pallidus gray matter volume |
| sri24thalamuslgm | Left thalamus gray matter volume |
| sri24thalamusrgm | Right thalamus gray matter volume |
| sri24heschllgm | Left transverse temporal gyri gray matter volume |
| sri24heschlrgm | Right transverse temporal gyri gray matter volume |
| sri24temporalsuplgm | Left superior temporal gyrus gray matter volume |
| sri24temporalsuprgm | Right superior temporal gyrus gray matter volume |
| sri24temporalpolesuplgm | Left superior temporal pole gray matter volume |
| sri24temporalpolesuprgm | Right superior temporal pole gray matter volume |
| sri24temporalmidlgm | Left middle temporal gyrus gray matter volume |
| sri24temporalmidrgm | Right middle temporal gyrus gray matter volume |
| sri24temporalpolemidlgm | Left middle temporal pole gray matter volume |
| sri24temporalpolemidrgm | Right middle temporal pole gray matter volume |
| sri24temporalinflgm | Left inferior temporal gyrus gray matter volume |
| sri24temporalinfrgm | Right inferior temporal gyrus gray matter volume |
| sri24cerebelumcrus1lvolume | Left crus i of cerebellar hemisphere volume |
| sri24cerebelumcrus1rvolume | Right crus i of cerebellar hemisphere volume |
| sri24cerebelumcrus2lvolume | Left crus ii of cerebellar hemisphere volume |
| sri24cerebelumcrus2rvolume | Right crus ii of cerebellar hemisphere volume |
| sri24cerebelum3lvolume | Left lobule iii of cerebellar hemisphere volume |
| sri24cerebelum3rvolume | Right lobule iii of cerebellar hemisphere volume |
| sri24cerebelum45lvolume | Left lobule iv, v of cerebellar hemisphere volume |
| sri24cerebelum45rvolume | Right lobule iv, v of cerebellar hemisphere volume |
| sri24cerebelum6lvolume | Left lobule vi of cerebellar hemisphere volume |
| sri24cerebelum6rvolume | Right lobule vi of cerebellar hemisphere volume |
| sri24cerebelum7blvolume | Left lobule viib of cerebellar hemisphere volume |
| sri24cerebelum7brvolume | Right lobule viib of cerebellar hemisphere volume |
| sri24cerebelum8lvolume | Left lobule viii of cerebellar hemisphere volume |
| sri24cerebelum8rvolume | Right lobule viii of cerebellar hemisphere volume |
| sri24cerebelum9lvolume | Left lobule ix of cerebellar hemisphere volume |
| sri24cerebelum9rvolume | Right lobule ix of cerebellar hemisphere volume |
| sri24cerebelum10lvolume | Left lobule x of cerebellar hemisphere (flocculus) volume |
| sri24cerebelum10rvolume | Right lobule x of cerebellar hemisphere (flocculus) volume |
| sri24vermis1gm | Anterior vermis gray matter volume |
| sri24vermis2gm | Posterior vermis gray matter volume |
| sri24vermis3gm | Inferior vermis gray matter volume |
| sri24cblmhemiwhtlwm | Cerebellum hemisphere white matter left volume |
| sri24cblmhemiwhtrwm | Cerebellum hemisphere white matter right volume |
| sri24ponswm | Pons white matter volume |
| sri24corpuscallosumwm | Corpus callosum white matter volume |
| sri24wm400wm400lwm | White matter volume within the left hemisphere of the subcortical white matter region (including the centrum semiovale) |
| sri24wm400wm400rwm | White matter volume within the right hemisphere of the subcortical white matter region (including the centrum semiovale) |
| sri24vtlslateralvtllcsf | Left lateral ventricle cerebral spinal fluid volume |
| sri24vtlslateralvtlrcsf | Right lateral ventricle cerebral spinal fluid volume |
| sri24vtlsthirdvtllcsf | Left third ventricle cerebral spinal fluid volume |
| sri24vtlsthirdvtlrcsf | Right third ventricle cerebral spinal fluid volume |
| sri24suptentsupratentoriumv | Suptent supratentorium volume |

**Table S4**. **Hyperparameters and their sampling priors of gradient boosted trees that were tuned with Bayesian optimization.** $LU(a, b)$ denotes a log-uniform distribution on the interval $[a, b]$.

| Hyperparameter | Sampling prior |
| --- | --- |
| Step size shrinkage | $LU({10}^{-5}, 1.25)$ |
| $L_{1}$ regularization strength | $LU({10}^{-6}, 4096)$ |
| $L_{2}$ regularization strength | $LU({10}^{-6}, 2048)$ |

**Table S5. Statistical comparison of confounder-adjusted vs. unadjusted AUROC values for FreeSurfer features.** For each disorder and both classifiers, the 150 confounder-adjusted and 150 unadjusted AUROC values obtained on the unpermuted data were tested for equal means via two-tailed Welch’s *t* test. The resulting *t* statistics and *p* values are given in the table. Significant *p* values (with significance level after Bonferroni correction of $\alpha_{adj}=\frac{0.05}{20}=0.0025$) are given in bold. **GBM-CCE:** Gradient boosting model classifier chain ensemble. **LRC:** Logistic regression classifier.

| **Disorder** | **Model** | ***t* statistic** | ***p* value** |
| --- | --- | --- | --- |
| Major Depressive Disorder | GBM-CCE | -4.7095 | **<0.0001** |
|  | LRC | -3.9612 | **0.0001** |
| Bipolar Disorder | GBM-CCE | -3.9054 | **0.0001** |
|  | LRC | -5.9030 | **<0.0001** |
| Psychotic Symptoms | GBM-CCE | 0.9394 | 0.3483 |
|  | LRC | -2.1024 | 0.0364 |
| ADHD | GBM-CCE | 12.2695 | **<0.0001** |
|  | LRC | -3.7785 | **0.0002** |
| Oppositional Defiant Disorder | GBM-CCE | 6.7230 | **<0.0001** |
|  | LRC | -4.8318 | **<0.0001** |
| Conduct Disorder | GBM-CCE | 8.0478 | **<0.0001** |
|  | LRC | -8.6011 | **<0.0001** |
| PTSD | GBM-CCE | 2.2782 | 0.0234 |
|  | LRC | -5.2683 | **<0.0001** |
| Obsessive Compulsive Disorder | GBM-CCE | -6.3649 | **<0.0001** |
|  | LRC | -5.8546 | **<0.0001** |
| Generalized Anxiety Disorder | GBM-CCE | -3.3526 | **0.0009** |
|  | LRC | -0.4799 | 0.6317 |
| Social Anxiety Disorder | GBM-CCE | -1.0563 | 0.2917 |
|  | LRC | 1.5944 | 0.1119 |

**Table S6. Statistical comparison of confounder-adjusted vs. unadjusted AUROC values for SRI24 features.** For each disorder and both classifiers, the 150 confounder-adjusted and 150 unadjusted AUROC values obtained on the unpermuted data were tested for equal means via two-tailed Welch’s *t* test. The resulting *t* statistics and *p* values are given in the table. Significant *p* values (with significance level after Bonferroni correction of $\alpha_{adj}=\frac{0.05}{20}=0.0025$) are given in bold. **GBM-CCE:** Gradient boosting model classifier chain ensemble. **LRC:** Logistic regression classifier.

| **Disorder** | **Model** | ***t* statistic** | ***p* value** |
| --- | --- | --- | --- |
| Major Depressive Disorder | GBM-CCE | -1.9550 | 0.0515 |
|  | LRC | -4.7534 | **<0.0001** |
| Bipolar Disorder | GBM-CCE | -3.8748 | **0.0001** |
|  | LRC | -5.0398 | **<0.0001** |
| Psychotic Symptoms | GBM-CCE | -2.1785 | 0.0302 |
|  | LRC | -1.1510 | 0.2506 |
| ADHD | GBM-CCE | -3.7112 | **0.0002** |
|  | LRC | -7.7085 | **<0.0001** |
| Oppositional Defiant Disorder | GBM-CCE | -4.2434 | **<0.0001** |
|  | LRC | -7.5022 | **<0.0001** |
| Conduct Disorder | GBM-CCE | 4.2552 | **<0.0001** |
|  | LRC | -0.9835 | 0.3261 |
| PTSD | GBM-CCE | -4.7036 | **<0.0001** |
|  | LRC | -6.4321 | **<0.0001** |
| Obsessive Compulsive Disorder | GBM-CCE | 3.9418 | **0.0001** |
|  | LRC | -1.3513 | 0.1776 |
| Generalized Anxiety Disorder | GBM-CCE | -0.7007 | 0.4840 |
|  | LRC | -3.5254 | **0.0005** |
| Social Anxiety Disorder | GBM-CCE | 0.9994 | 0.3184 |
|  | LRC | -1.1858 | 0.2367 |

1. <https://nda.nih.gov/data_structure.html?short_name=btsv01> [↑](#footnote-ref-2)
